# A Flexible Binding Site Architecture Provides New Insights into CcpA Global Regulation in Gram-Positive Bacteria

**DOI:** 10.1128/mBio.02004-16

**Published:** 2017-01-24

**Authors:** Yunpeng Yang, Lu Zhang, He Huang, Chen Yang, Sheng Yang, Yang Gu, Weihong Jiang

**Affiliations:** a Key Laboratory of Synthetic Biology, Institute of Plant Physiology and Ecology, Shanghai Institutes for Biological Sciences, Chinese Academy of Sciences, Shanghai, China; b University of Chinese Academy of Sciences, Beijing, China; c Jiangsu National Synergetic Innovation Center for Advanced Materials, SICAM, Nanjing, China; d Shanghai Collaborative Innovation Center for Biomanufacturing Technology, Shanghai, China; Korea Advanced Institute of Science and Technology

## Abstract

Catabolite control protein A (CcpA) is the master regulator in Gram-positive bacteria that mediates carbon catabolite repression (CCR) and carbon catabolite activation (CCA), two fundamental regulatory mechanisms that enable competitive advantages in carbon catabolism. It is generally regarded that CcpA exerts its regulatory role by binding to a typical 14- to 16-nucleotide (nt) consensus site that is called a catabolite response element (*cre*) within the target regions. However, here we report a previously unknown noncanonical flexible architecture of the CcpA-binding site in solventogenic clostridia, providing new mechanistic insights into catabolite regulation. This novel CcpA-binding site, named *cre*_var_, has a unique architecture that consists of two inverted repeats and an intervening spacer, all of which are variable in nucleotide composition and length, except for a 6-bp core palindromic sequence (TGTAAA/TTTACA). It was found that the length of the intervening spacer of *cre*_var_ can affect CcpA binding affinity, and moreover, the core palindromic sequence of *cre*_var_ is the key structure for regulation. Such a variable architecture of *cre*_var_ shows potential importance for CcpA’s diverse and fine regulation. A total of 103 potential *cre*_var_ sites were discovered in solventogenic* Clostridium acetobutylicum*, of which 42 sites were picked out for electrophoretic mobility shift assays (EMSAs), and 30 sites were confirmed to be bound by CcpA. These 30 *cre*_var_ sites are associated with 27 genes involved in many important pathways. Also of significance, the *cre*_var_ sites are found to be widespread and function in a great number of taxonomically different Gram-positive bacteria, including pathogens, suggesting their global role in Gram-positive bacteria.

## IMPORTANCE

In Gram-positive bacteria, the global regulator CcpA controls a large number of important physiological and metabolic processes. Although a typical consensus CcpA-binding site, *cre*, has been identified, it remains poorly explored for the diversity of CcpA-mediated catabolite regulation. Here, we discovered a novel flexible CcpA-binding site architecture (*cre*_var_) that is highly variable in both length and base composition but follows certain principles, providing new insights into how CcpA can differentially recognize a variety of target genes to form a complicated regulatory network. A comprehensive search further revealed the wide distribution of *cre*_var_ sites in Gram-positive bacteria, indicating it may have a universal function. This finding is the first to characterize such a highly flexible transcription factor-binding site architecture, which would be valuable for deeper understanding of CcpA-mediated global catabolite regulation in bacteria.

## INTRODUCTION

Carbon catabolite repression (CCR) and carbon catabolite activation (CCA) are two of the most fundamental regulatory mechanisms in microbes (1, 2), enabling them to adapt quickly to environmental changes. In Gram-positive bacteria, the master regulator mediating CCR and CCA is catabolite control protein A (CcpA), a protein of the LacI-GalR family (3). CcpA is a pleiotropic regulator involved in many important cellular processes, including bacterial pathogenicity (4–6).

It is known that CcpA executes its regulation via binding to a so-called catabolite-responsive element (*cre*) within the promoter or protein-coding regions of the target genes (7). The consensus sequence of *cre* has been determined to be TGWAANCGNTNWCA in *Bacillus subtilis*, a model organism of Gram-positive bacteria, in which N represents any base and W represents A or T (8). Additional *cre*s identified later in *B. subtilis* also closely match this consensus sequence (7, 9). However, it has recently been found that, in some cases, CcpA employed two different binding motifs (one is a typical *cre* and the other one is an atypical *cre*) to regulate the central carbon metabolism (10). This indicates that the general understanding of CcpA activity is superficial and the mechanism by which CcpA exerts its regulation is more sophisticated than we know.

Solventogenic clostridia are of great interest because they are able to produce a series of bulk chemicals (11), among which *n*-butanol and ethanol are important liquid fuels. Our research group previously revealed a core *cre* consensus sequence, WTGWAAACGWTWWCAW (where W represents A or T) that is responsible for CcpA binding in *Clostridium acetobutylicum*, a typical species of solventogenic clostridia (12). This sequence is highly similar to that of *B. subtilis*, but meanwhile, a large number of genes that exhibited greatly altered transcription after *ccpA* inactivation do not contain the *cre* sites in their promoter or protein-coding region (12), thus raising the question as to whether there exist atypical binding sites recognized by CcpA.

Here, we identified a novel flexible architecture of binding sites recognized by CcpA. This binding motif was then shown to be widespread in Gram-positive bacteria, indicating its importance in CcpA-mediated regulation. Based on these results, we identify a large number of new target genes controlled by CcpA and thereby chart a more complete CcpA regulatory network in *C. acetobutylicum*.

## RESULTS

### Discovery of novel transcriptional binding sites for CcpA regulation.

**FIG 1:**
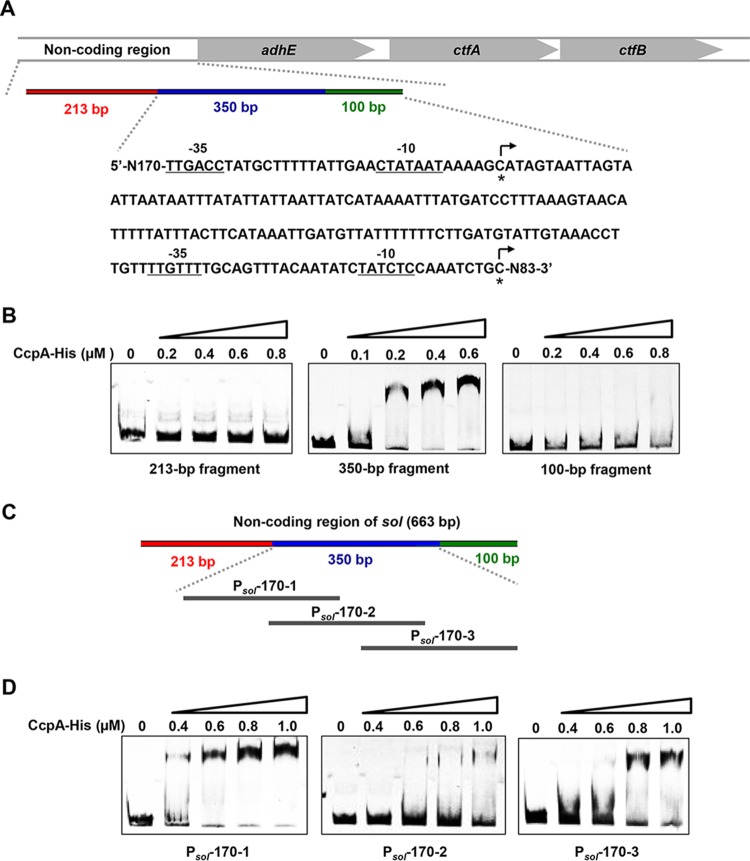
Functional analysis of the CcpA-binding sites in the noncoding region of the *sol* operon. (A) The noncoding region of the* sol* operon was divided into three fragments (213, 350, and 100 bp). The −10 region and −35 region are underlined. The two transcription start sites (13) are indicated by bent arrows. (B) EMSAs of His_6_-CcpA binding to the 213-, 350-, and 100-bp fragments labeled with Cy5. (C) The 350-bp fragment of *sol* was further divided into three 170-bp fragments (P_*sol*_-170-1, P_*sol*_-170-2, and P_*sol*_-170-3). (D) EMSAs of His_6_-CcpA binding to P_*sol*_-170-1, P_*sol*_-170-2 and P_*sol*_-170-3.

Based on our previously performed comparative transcriptomic analysis (12), we found that, among a total of 1,394 genes that showed greatly altered transcription after *ccpA* inactivation, only 154 genes contain the typical *cre* (WTGWAAACGWTWWCAW [W represents A or T]) sites within their promoter or coding region. Thus, the presence of noncanonical CcpA-binding sites within these genes is strongly suggested. To explore this possibility, we chose *sol* (CAP0162-0164), a key operon responsible for acid reassimilation and solvent formation in *C. acetobutylicum* (13), containing no typical *cre* sites but showing high binding affinity to CcpA (12), for a detailed examination. In the first step, a 663-bp promoter region (−663 to −1 bp relative to the translational start point) of *sol* was divided into three fragments (213, 350, and 100 bp) for electrophoretic mobility shift assays (EMSAs) (Fig. 1A). A strongly shifted band was observed for the 350-bp fragment (Fig. 1B), suggesting the existence of binding sites within this region. Next, this 350-bp fragment was further divided into three 170-bp segments (P_*sol*_-170-1, P_*sol*_-170-2, and P_*sol*_-170-3), with 80 bp overlapping one by one, for EMSAs (Fig. 1C). Interestingly, a DNA bind shift was observed for all three segments (Fig. 1D), implying that there may exist more than one CcpA-binding site within the promoter region of *sol*.

**FIG 2:**
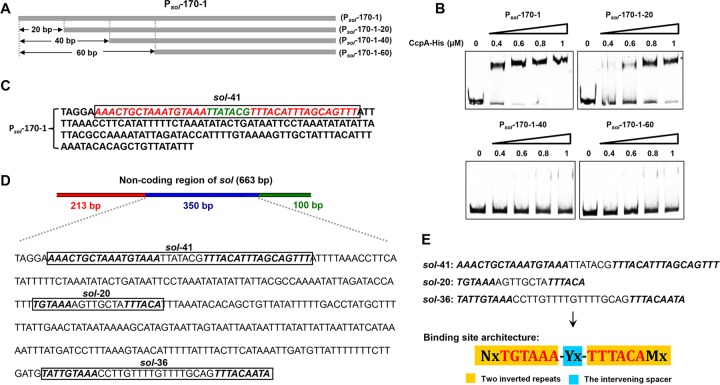
Identification of novel CcpA-binding sites in *C. acetobutylicum*. (A) Truncation of P_*sol*_-170-1. Sequences 20, 40, and 60 bp in length were cut off from P_*sol*_-170-1, yielding three truncated fragments (P_*sol*_-170-1-20, P_*sol*_-170-1-40, and P_*sol*_-170-1-60). (B) EMSAs of His_6_-CcpA binding to P_*sol*_-170-1 and the three truncated fragments. His_6_-CcpA concentrations of 0 to 1.0 µM were used. (C) Mining of a potential CcpA-binding site in P_*sol*_-170-1 via visual analysis. The putative CcpA-binding site is represented by a box. The two inverted repeats and the intervening spacer of the bind site are colored red and green. (D) Three putative CcpA-binding sites (*sol-*41, *sol-*20, and *sol-*36) found in the 350-bp noncoding region of *sol*. These three sites share two conserved inverted repeats (TGTAAA/TTTACA), which are highlighted in boldface and italics. (E) A common sequence (N_*x*_TGTAAA-Y_*x*_-TTTACAM_*x*_) was derived from these three CcpA-binding sites. N and M represent any bases complementary to each other, Y represents any base, and *x* represents the number of the bases included in the two inverted repeats and intervening spacer, which was variable.

To confirm this hypothesis, P_*sol*_-170-1, which had the strongest shifted signal among these three segments, was gradually truncated, and the resulting three truncated fragments, namely, P_*sol*_-170-1 minus 20, 40, and 60 bp, respectively, were examined (Fig. 2A). The results showed that the affinity of P_*sol*_-170-1 for CcpA was almost completely abolished with a 40- or 60-bp deletion (Fig. 2B), suggesting a binding site overlapping or within the deleted region. Encouragingly, visual scanning of this 60-bp region identified a 41-nucleotide (nt) palindromic sequence (AAACTGCTAAATGTAAA-TTATACG-TTTACATTTAGCAGTTT) comprising two 17-nt inverted repeats separated by 7 nt (Fig. 2C). According to the characteristics of this palindromic sequence, we further found two other similar palindromic sequences within the 350-bp fragment, which harbor 6- and 9-nt inverted repeats separated by 8 and 18 nt, respectively (Fig. 2D). A common feature of these three palindromic sequences (designated *sol-*41, *sol-*20, and *sol-*36, respectively) is the two repeats that contain the core palindromic sequence TGTAAA/TTTACA, as well as the intervening spacer region; the difference is the variable length of the two repeats and the intervening spacer region. Thus, this yielded the architecture N_*x*_TGTAAA-Y_*x*_-TTTACAM_*x*_ (where Y represents any base, N and M also represent any base but are complementary to each other, and *x* represents the base number) (Fig. 2E). Compared with the known *cre* consensus in bacteria such as *Bacillus* (14), *Lactobacillus* (15), and *Staphylococcus* species (16), this binding site architecture is quite distinct, which is an inverted TA-rich sequence separated by a variable (length and nucleotide) spacer region. This architecture is different from all known CcpA-binding *cre* motifs, which are normally 14 or 16 bp in length, including several highly conserved nucleotides (7, 17–20). We named this novel flexible CcpA binding site architecture “*cre*_var_”.

### Wide occurrence of the novel binding site *cre*_var_ in *C. acetobutylicum*.

**FIG 3:**
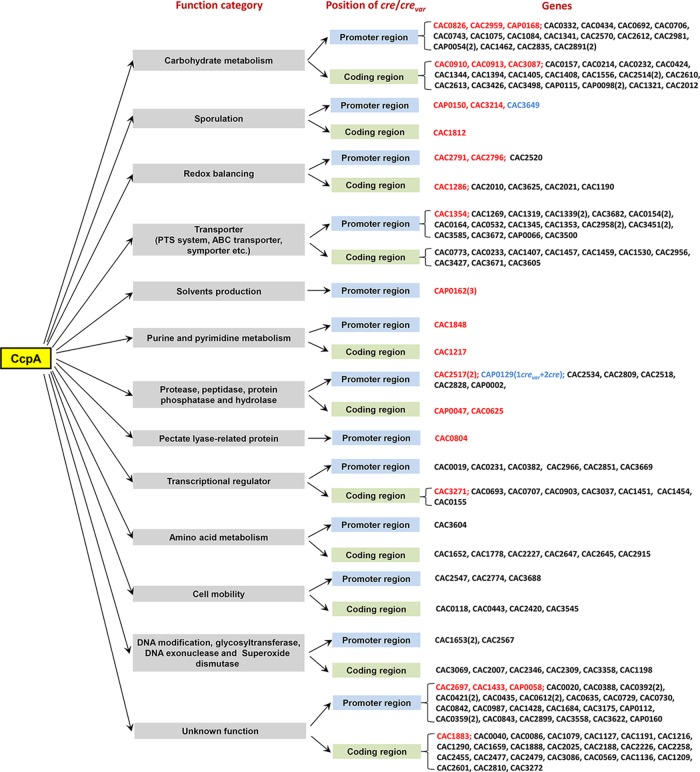
Overview of the genes affected by CcpA. The genes that have been confirmed to be directly regulated by CcpA (from EMSA data in Fig. S1) via *cre*_var_ sites are shown in red; the *cre*-associated genes that were predicted to be significantly affected by CcpA (>2-fold transcriptional change after *ccpA* inactivation) according to our previous work (12) are shown in black; and the genes associated with both *cre* and *cre*_var_ and also showing significant transcriptional changes (>2-fold) after *ccpA* inactivation are shown in blue. The genes associated with multiple *cre* or *cre*_var_ sites are annotated with the number of sites given in parentheses. CAC, genes located in chromosome of *C. acetobutylicum*; CAP, genes located in the megaplasmid of *C. acetobutylicum*.

**FIG 4:**
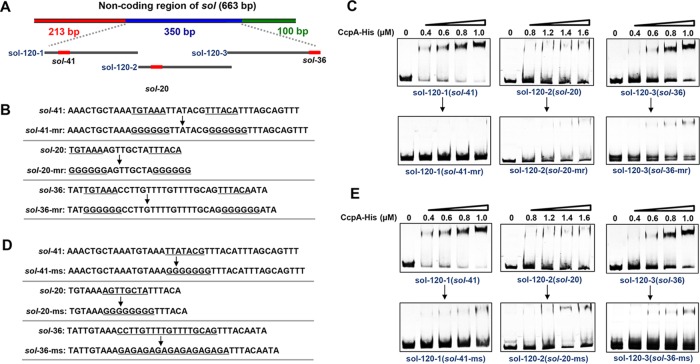
Mutational analysis of the *cre*_var_ sites in the noncoding region of *sol*. (A) The 350-bp sequence containing the three putative* cre*_var_ sites was divided into three 120-bp fragments (sol-120-1, sol-120-2, and sol-120-3), each of which harbored one *cre*_var_ site (*sol-*41, *sol-*20, and *sol-*36), which is represented by a red box. (B) Mutation of the two inverted repeats of the three *cre*_var_ sites. The two 6-nt repeats of *sol-*41, *sol-*20, and *sol-*36 were all mutated to GGGGGG, yielding *sol-*41-mr, *sol-*20-mr, and *sol-*36-mr, respectively. (C) EMSAs of His_6_-CcpA binding to the three 120-bp fragments as well as their derived fragments mutated in the two repeats. (D) Mutation of the intervening spacer of each of the three *cre*_var_ sites (*sol-*41-ms, *sol-*20-ms, and *sol-*36-ms). (E) EMSAs of His_6_-CcpA binding to the three 120-bp fragments as well as their derived fragments mutated in the intervening spacers.

To explore the distribution of the *cre*_var_ sites in *C. acetobutylicum*, we performed a genome-wide scan using the RegPredict web server (21), in which the two 6-nt repeats of *cre*_var_ were fixed but the length of the intervening region was variable, covering 0 to 40 nt. The search result revealed 103 potential *cre*_var_ sites that belong to 99 genes (see Table S1 in the supplemental material). Next, 42 (the associated genes showed ≥2-fold transcriptional changes after *ccpA* inactivation) (12) of these 103 potential *cre*_var_ sites were picked out for EMSAs to examine the quality of the prediction result. Finally, 30 *cre*_var_ sites were confirmed to be bound by CcpA, including the above-mentioned three *cre*_var_ sites in the upstream region of *sol* (see Fig. 4C) and the other 27 *cre*_var_ sites that are associated with 26 genes (see Fig. S1 in the supplemental material). Among these 30 *cre*_var_ sites, 20 sites are located in promoter regions, whereas 10 sites are inside protein-coding regions (see Table S2 in the supplemental material). The majority of these *cre*_var_-associated genes can be grouped into certain functional subsets (Fig. 3). Several genes are involved in important bioprocesses, including substance transport and metabolism, redox balancing, sporulation, and solvent production. Besides, by comparing the distribution of *cre* and *cre*_var_ in the genome, *cre*_var_ sites were found to be predominantly associated with genes belonging to certain function categories, i.e., sporulation, solvent production, and purine and pyrimidine metabolism (Fig. 3), which suggests that *cre*_var_ may play more important roles in CcpA regulation of these genes, thus enabling a more comprehensive regulatory network of CcpA.

10.1128/mBio.02004-16.1 TABLE S1  Putative CcpA-binding *cre*_var_ sites in the chromosome of *C. acetobutylicum*. Download TABLE S1, DOCX file, 0.03 MB. Copyright © 2017 Yang et al. 2017 Yang et al. This content is distributed under the terms of the Creative Commons Attribution 4.0 International license.

10.1128/mBio.02004-16.2 FIG S1  Verification of new CcpA regulatory targets containing *cre*_var_. Shown are results from EMSAs of CcpA binding to 26 oligonucleotides containing putative *cre*_var_ sites in* C. acetobutylicum*. The oligonucleotide derived from the promoter region of *thl* (CAC2873) was used as the negative control. The promoter sequence of CAC2517 contains two putative* cre*_var_ sites. Download FIG S1, DOCX file, 0.4 MB. Copyright © 2017 Yang et al. 2017 Yang et al. This content is distributed under the terms of the Creative Commons Attribution 4.0 International license.

10.1128/mBio.02004-16.3 TABLE S2  CcpA-binding *cre*_var_ sites identified in *C. acetobutylicum*. Download TABLE S2, DOCX file, 0.02 MB. Copyright © 2017 Yang et al. 2017 Yang et al. This content is distributed under the terms of the Creative Commons Attribution 4.0 International license.

### Characterization of the binding motif *cre*_var_.

Next, we attempted to assess the importance of the signature sequences of *cre*_var_ for CcpA binding. The two inverted repeats and the intervening spacers of the three *cre*_var_ sites (*sol-*41, *sol-*20, and *sol-*36) mentioned above were mutated (Fig. 4A, B, and D), and then the binding activities of CcpA with the three mutated 120-bp sequences were determined. The results showed that the mutations at two repeats completely abolished the binding of CcpA to *sol-*41, whereas a light binding to *sol-*20 and *sol-*36 was maintained (Fig. 4C). In contrast, mutations in the intervening region weakened, to different extent, the binding affinities of CcpA to *sol-*41, *sol-*20, and *sol-*36 (Fig. 4E). These findings suggest that both the two inverted repeats and intervening spacer are crucial for CcpA-*cre*_var_ binding.

In addition to *in vitro* experiments, we also examined CcpA binding to *cre*_var_ sites *in vivo* by using a reporter gene. As shown in Fig. S2 in the supplemental material, the LacZ activity assay revealed that single mutation of either the *sol-*41 or *sol-*20 site and mutation of both the *sol-*41 and *sol-*20 sites resulted in significantly decreased strength of promoter P_*sol*_ in the wild-type strain, whereas no significant difference was observed in the 824*ccpA* strain (in which *ccpA* was disrupted). This further confirmed that *sol-*41 and *sol-*20 are the CcpA-binding sites.

10.1128/mBio.02004-16.4 FIG S2  Validation of CcpA-binding sites in the noncoding region of the *sol* operon. (A) The mutation of *sol*-41 and *sol*-20 in the promoter region of the *sol* operon. *sol*-41 and *sol*-20 are mutated separately or simultaneously. P_*sol*_ and its mutated derivatives are cloned into the *lacZ* reporter plasmid. (B) β-Galactosidase assay of the plasmids constructed in panel A at 69 h in both the *C. acetobutylicum* wild-type strain (824) and *ccpA*-inactivated (824*ccpA*) strain. Means and standard deviations were from two independent biological replicates (***, *P* ≤ 0.001, **, *P* ≤ 0.01, and *, *P* ≤ 0.05, Student’s *t* test). Download FIG S2, DOCX file, 0.1 MB. Copyright © 2017 Yang et al. 2017 Yang et al. This content is distributed under the terms of the Creative Commons Attribution 4.0 International license.

**FIG 5:**
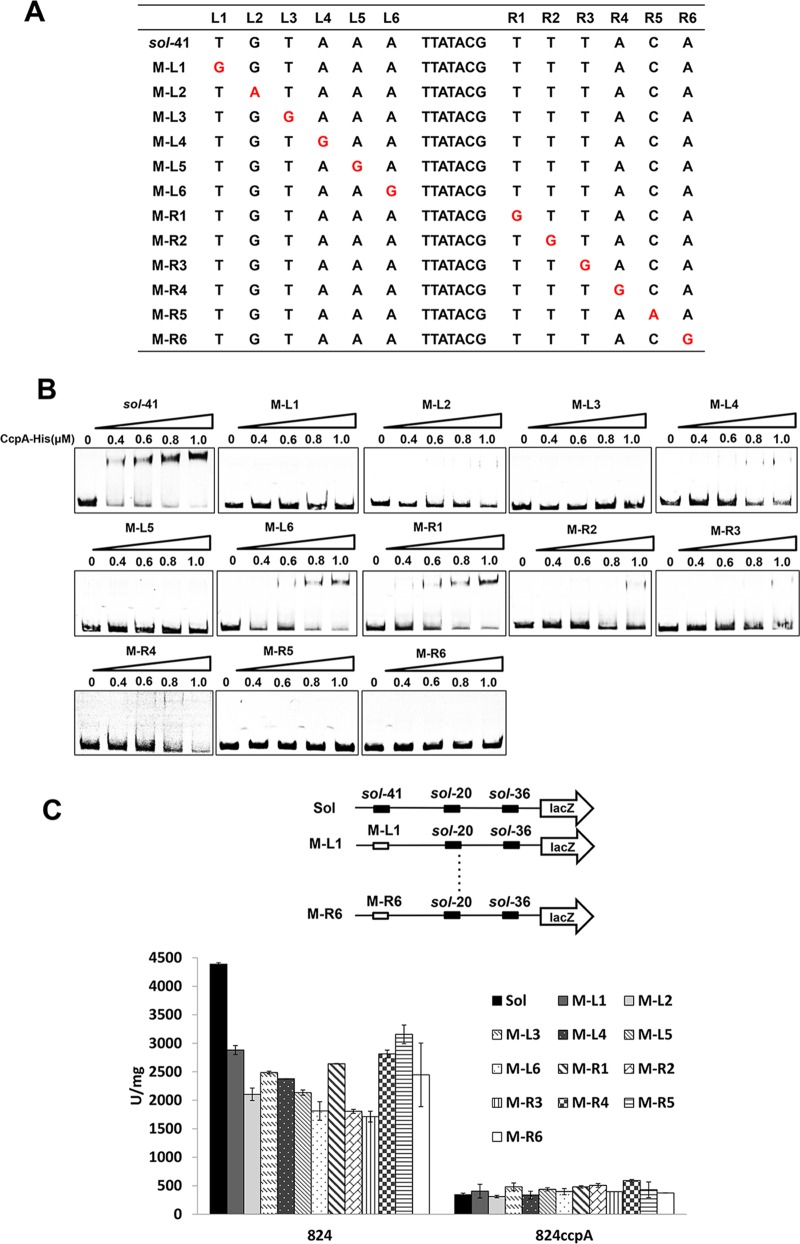
Characterization of the novel CcpA-binding sites in *C. acetobutylicum*. (A) Single point mutation of the inverted repeats of *sol-*41. The mutation site is marked in red. (B) EMSAs of His_6_-CcpA binding to sol-120-1 containing *sol*-41 and its mutated derivatives. Concentrations of 0 to 1.0 µM of His_6_-CcpA were used. (C) *In vivo* assay of P_*sol*_ and its derivatives in both the *C. acetobutylicum* wild-type and *ccpA*-inactivated strain. The data represent the average from two independent samples.

Specific to the two inverted 6-nt repeats, since they were important for CcpA-*cre*_var_ binding, we attempted to examine whether each nucleotide is essential. Thus, each nucleotide in the two 6-nt repeats of *sol-*41 was separately mutated, yielding 12 derivative probes for EMSA analysis (Fig. 5A). The EMSA results showed that single mutation of each one of the outer five nucleotides (L1, L2, L3, L4, and L5 or R2, R3, R4, R5, and R6) thoroughly eliminated CcpA–*sol-*41 binding, while mutation of L6 or R1 still retained a slight binding (Fig. 5B). Next, the *in vivo* experiments using a *lacZ* reporter were performed to see the strength variations between P_*sol*_ and its 12 derivatives. While no significant difference was observed in 824*ccpA* (the control with *ccpA* disruption), all 12 single mutations resulted in greatly decreased LacZ activity in the wild-type strain (Fig. 5C), which are consistent with the *in vitro* EMSA results. These findings demonstrate that each nucleotide in the two 6-nt arms of *cre*_var_ is important for CcpA binding.

### The variable intervening region length of *cre*_var_ sites affects CcpA binding affinity.

**FIG 6:**
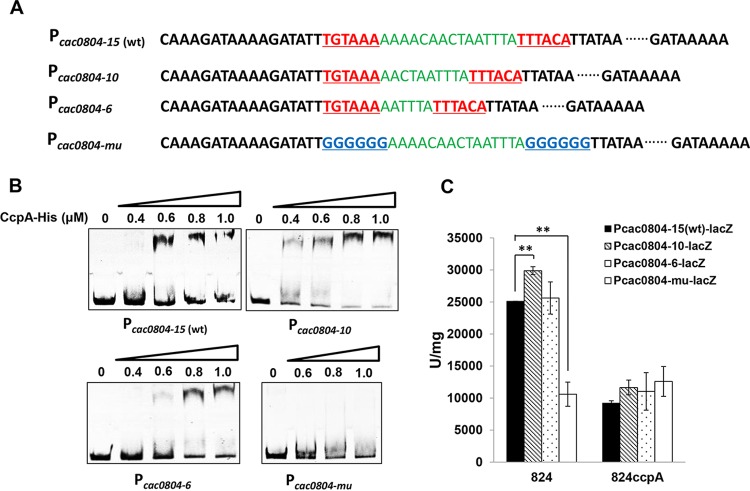
Influence of the intervening spacer length within *cre*_var_ on CcpA regulation. (A) Construction of artificial promoters harboring the *cre*_var_ sites with different spacer lengths. The two inverted repeats and the intervening spacer are highlighted with red and green, respectively. P_*cac0804-15*(wt)_ is the natural promoter sequence of the *cac0804* gene. P_*cac0804-*mu_ was constructed as a negative control by mutating the two inverted repeats of P_*cac0804-15*(wt)_. (B) EMSAs of His_6_-CcpA binding to P_*cac0804-15*(wt)_, P_*cac0804-10*_, P_*cac0804-6*_, and P_*cac0804-*mu_. (C) *In vivo* assay of CcpA regulation of P_*cac0804-15*(wt)_ and its derivatives in both the *C. acetobutylicum* wild-type strain (824) and *ccpA*-inactivated strain (824*ccpA*). Means and standard deviations were from two independent biological replicates (**, *P* ≤ 0.01, Student’s *t* test).

Since the intervening spacer of *cre*_var_ is variable, the question arose as to whether the spacer length affected CcpA-DNA binding. We explored this possibility by using promoter P_*cac0804-15*(wt)_, which contains a 27-nt *cre*_var_ site with a 15-nt intervening spacer (Fig. 6A). Here, the major reason for choosing P_*cac0804-15*(wt)_ for investigation is that, among the 15 genes identified to contain a sole *cre*_var_ site in their promoter regions (Fig. 3), the *cac0804* gene was the only one showing steady and significant upregulation (over 2-fold) in transcriptional level after *ccpA* overexpression (data not shown), indicating a high CcpA binding affinity to the *cre*_var_ site in P_*cac0804-15*(wt)_. When this 15-nt spacer was truncated to 10 nt, a significantly altered CcpA-DNA binding affinity occurred (Fig. 6B and C); further truncated to 6 nt, no binding affinity changes were found between P_*cac0804-6*_ and P_*cac0804-15*(wt)_ (Fig. 6C). In contrast, for the *ccpA*-inactivated strain (used here as a control), no significant differences in LacZ expression were observed after truncation of the spacer (Fig. 6C). These results suggest that the intervening spacer length within *cre*_var_ sites can influence CcpA-*cre*_var_ binding affinity.

### The *cre*_var_ sites are widely distributed in Gram-positive bacteria.

Because the *cre*_var_ sites occurred frequently in the *C. acetobutylicum* genome, we are curious whether this *cis* element is also present in other bacteria. To this end, we performed genome-wide searches in the classes *Clostridia* and *Bacilli*, two large groups in Gram-positive bacteria. Surprisingly, the *cre*_var_ sites were found in the genome of several members of these two classes, including pathogens, and were especially abundant in *Clostridium* and *Bacillus* species, in which over 100 *cre*_var_ sites were predicted to be present in *Clostridium acetobutylicum*, *Clostridium cellulolyticum*, *Clostridium difficile*, and *Bacillus cereus* (see Table S3 in the supplemental material). Importantly, like those identified in *C. acetobutylicum*, the *cre*_var_ sites present in these species also exhibited high diversity in the two inverted repeats and intervening spacer regions (Table S3). To our knowledge, only very few proteins have been found capable of recognizing DNA sequence separated by a variable spacer (22–25); however, *cre*_var_-like binding motifs that contain such a highly flexible spacer region have not been reported.

10.1128/mBio.02004-16.5 TABLE S3  Putative *cre*_var_ sites in class *Clostridia* and class *Bacilli*. Download Table S3, DOCX file, 0.1 MB. Copyright © 2017 Yang et al. 2017 Yang et al. This content is distributed under the terms of the Creative Commons Attribution 4.0 International license.

**FIG 7:**
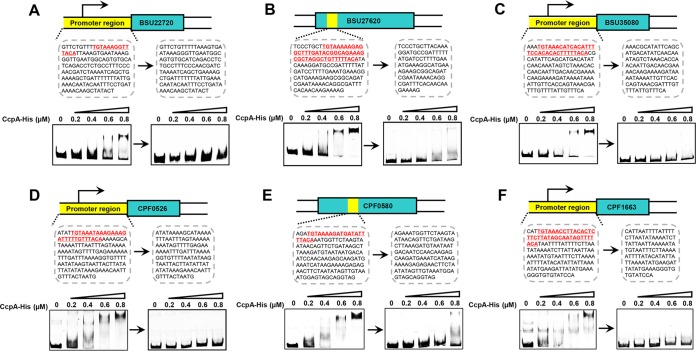
EMSA verification of CcpA binding to the putative *cre*_var_ sites in *B. subtilis* and *C. perfringens*. The sequences highlighted with red indicate the putative *cre*_var_ sites. *B. subtilis* CcpA and *C. perfringens* CcpA were purified and used for functional analysis of the *cre*_var_ sites in *B. subtilis* and *C. perfringens*, respectively.

We chose five potential *cre*_var_ sites from both *B. subtilis* (BSU10020, BSU14580, BSU22720, BSU27620, and BSU35080) and *C. perfringens* (CPF0042, CPF0484, CPF0526, CPF0580, and CPF1663) for EMSA verification. *B. subtilis* CcpA and *C. perfringens* CcpA were purified and used for functional analysis of the *cre*_var_ sites in *B. subtilis* and *C. perfringens*, respectively. Encouragingly, among these candidates, a substantial DNA band shift was observed for six *cre*_var_ sites (BSU22720, BSU27620, BSU35080, CPF0526, CPF0580, and CPF1663) (Fig. 7), indicating a high reliability of the predicted *cre*_var_ sites in the classes *Clostridia* and *Bacilli*.

## DISCUSSION

As an important regulator in Gram-positive bacteria, CcpA has remained little understood with respect to its pleiotropic regulatory function. This study has expanded CcpA’s target genes to a broader range in clostridia as well as some other Gram-positive bacteria, thereby providing new insights into CcpA regulation. Importantly, identification of the novel *cre*_var_ sites revealed a flexible binding site architecture used by CcpA to regulate its target genes. The variation in both the intervening spacer region and two inverted repeats of this *cre*_var_ motif, as well as its widespread occurrence in Gram-positive bacteria, suggests a more complex CcpA regulation than was previously understood.

To date, only very few proteins have been found capable of recognizing repeats separated by a variable spacer. As an example, the *Escherichia coli* cyclic AMP (cAMP) receptor protein (CRP)-binding sites contain a 6- or 8-bp spacer (22); additionally, the *E. coli* CytR repressor, with the assistance of the CRP, can recognize two inverted repeats separated by 10 to 13 bp or direct repeats separated by 1 bp (23). A latest example is the *E. coli* transcription factor HipB, which can recognize palindromic sequences with variable intervening spacer regions (24); moreover, the crystal structures of the HipB-HipA-*hipBA* promoter complex showed that HipBA binding to DNA with a long spacer can be achieved by DNA extrusion (25).

Here, the variation in the *cre*_var_ sites suggests diversity in the CcpA-binding sites for both repressed and activated target genes. The most distinct feature of *cre*_var_ is its intervening spacer region, which is flexible in both length (0- to 40-nt span) and base composition. This feature makes *cre*_var_ quite different from all known CcpA-binding *cre* motifs, which are normally 14 or 16 bp in length, including several highly conserved nucleotides (7, 17–20). For typical *cre* sites, the base variations may cause them to display different bend angles during CcpA binding; CcpA is also able to adjust its conformation to meet the changes in target DNA (7). However, such changes in binding angle appeared insufficient to affect the affinity of the DNA for CcpA (7). In contrast, for the atypical *cre*_var_, the spacer within its motif is variable in both length and base composition, which may cause greater changes in CcpA conformation during its binding to the targets.

Given the wide variation of the *cre*_var_ sites as well as the coexistence of *cre*_var_ and *cre*, we propose that this variability may be an effective mechanism for the diverse regulation of CcpA in Gram-positive bacteria. First, the variable spacer might affect the binding affinity of CcpA for its targets, which would enable CcpA to produce diverse regulatory outputs. For example, the regulation of *E. coli* CytR, a regulator belonging to the LacI family, was affected by artificially altering the half-site spacing in its binding sites, and the maximum changes in CytR regulation occurred in the short spacing variants (26). Second, the coexistence of *cre*_var_ and *cre* suggests a complementary or independent role of *cre*_var_ relative to *cre* in CcpA regulation, which would confer more choices to CcpA during its regulation. At least in some cases, CcpA may require more than one binding site to exert sophisticated gene regulation. For example, it has been found that CcpA employed two different binding motifs (WWGAAARCGYTTTCWW and TTTTYHWDHHWWTTTY) to regulate the central carbon metabolism in *Streptococcus suis* (10); besides, *cre*_var_ sites were predominantly found to be related to genes of certain function categories (Fig. 3), indicating a more important role of *cre*_var_, rather than *cre*, in CcpA regulation of these genes.

Another interesting finding here is that the widespread occurrence of the *cre*_var_ sites in Gram-positive bacteria, especially classes *Clostridia* and *Bacilli*. Using *B. subtilis* as an example, many essential genes involved in core metabolism were shown to be controlled by CcpA via *cre*_var_ (Table S3), although the predicted *cre*_var_ sites appeared to be fewer than the typical *cre* sites, which were previously estimated to number over 100 in *B. subtilis* (9, 27). The validation experiments in *B. subtilis* also supported this finding (Fig. 7). More importantly, the *cre*_var_ sites were found to be associated with several essential genes in the pathogenic bacteria, such as genes responsible for the phosphotransferase (PTS) system, cell motility and division, DNA replication and mismatch repair, and sporulation (Table S3). Of note, the *cre*_var_ sites were also present in the promoter or protein-coding regions of certain toxin or virulence genes, e.g., the* texT* gene in *Clostridium tetani* (28) and a possible virulence factor gene (SE0184) in *Staphylococcus epidermidis* (29) (Table S3). All of these findings further suggest the potential importance of *cre*_var_ as a* cis* element.

It should be noted for the two 6-nt-sequence core region that although this sequence appears to be conserved in the *cre*_var_ motif, we cannot exclude the possibility that the sequence is also changeable to a certain extent without impacting CcpA recognition of the targets. In this study, we used the common sequence (TGTAAA-Y_*x*_-TTTACA) that was extracted from the three binding sites upstream of the *sol* genes as a template, in which the two inverted repeats were fixed. Apparently, using such a template to search for more CcpA-binding sites has limited the 6-nt core region of the repeats; thus, the yielded binding sites do not reflect all the potential variations in this region. To determine the occurrence frequency of each base at each location of this 6-bp inverted repeat, the strategy such as chromatin immunoprecipitation followed by high-throughput sequencing (chromatin immunoprecipitation sequencing [ChIP-seq]) should be useful. This study is under way.

In summary, we have discovered an unrealized highly flexible architecture of CcpA-binding sites. The motif *cre*_var_, which is variable in both the two repeats and the intervening spacer region, provides new insight into the structure of CcpA recognition sites in Gram-positive bacteria. Such a variation of *cre*_var_ may provide an effective means to CcpA for fine-tuning the regulatory network. Given the wide distribution of the *cre*_var_ in Gram-positive bacteria, it is conceivable that this flexible motif plays an important role in CcpA-mediated regulation of cellular properties.

## MATERIALS AND METHODS

### Strains and plasmid construction.

The strains and plasmids used in this work are listed in Table S4 in the supplemental material. To express the CcpA protein of *C. acetobutylicum*, *ccpA* (CAC3037) was PCR amplified and cloned into pET-28a (Novagen, Madison, WI), yielding the plasmid pET-28a-ccpAcac. Similarly, pET-28a-ccpAbsu and pET-28a-ccpAcpf were constructed to express CcpA from *Bacillus subtilis* and *Clostridium perfringens*. pET-28a-HPrK and pGEX4T1-HPr were used for HPr kinase (HPrK) and HPr expression (30). P_*sol*_ and P_*cac0804*_ and their derivatives were PCR amplified and cloned into pIMP1-lacZ (31) for β-galactosidase assays.

10.1128/mBio.02004-16.6 TABLE S4  Strains and plasmids used in this study. Download Table S4, DOCX file, 0.03 MB. Copyright © 2017 Yang et al. 2017 Yang et al. This content is distributed under the terms of the Creative Commons Attribution 4.0 International license.

### Media and cultivation conditions.

*Escherichia coli* was grown in Luria-Bertani (LB) medium at 37°C with the addition of chloramphenicol (Chloromycetin [25 µg/ml]), kanamycin (50 µg/ml), ampicillin (100 µg/ml), and spectinomycin (50 µg/ml) when needed. *C. acetobutylicum* was cultivated in CGM medium (32) and P2 medium (33). Thiamphenicol (8 µg/ml) were added to the P2 medium when needed.

### Identification of CcpA-binding sites.

The RegPredict web server (21) was used to search all potential *cre*_var_ sites on the genome of *C. acetobutylicum* based on the architecture of the template TGTAAA-Y_*x*_-TTTACA (Yx ranged from 0 to 40 nt). The search regions cover nucleotide positions from −500 to +2000 relative to the translational start sites of all the genes. The results were further artificially analyzed to eliminate the redundant data.

### Protein overexpression and purification.

The His_6_-tagged CcpA and HPrK and glutathione *S*-transferase (GST)-tagged HPr were expressed and purified as described previously (12, 30). The purified proteins were checked by SDS-PAGE.

### EMSAs.

The DNA probes used in EMSAs were generated as follows. First, the unlabeled DNA fragments were amplified from the genome using specific primer pairs containing a universal sequence (5′ AGCCAGTGGCGATAAG 3′) at the 5′ terminal. Second, the DNA fragments were Cy5 labeled by PCR using the universal primer 5′ AGCCAGTGGCGATAAG 3′, with Cy5 labeled at the 5′ end. Finally, the resulting Cy5-labeled probes were recovered by agarose gel electrophoresis.

The EMSAs with *C. acetobutylicum* CcpA were performed as described previously (30). The EMSAs with *B. subtilis* CcpA and *C. perfringens* CcpA were performed similarly, except that the phosphorylated Hpr was not used.

### β-Galactosidase assays.

The *C. acetobutylicum* strains harboring the plasmids pIMP1-P_*sol*_-*lacZ*, pIMP1-P_*cac0804*_-*lacZ*, and their derivatives (listed in Table S4) were grown in P2 medium containing 60 g/liter glucose as the sole carbon source. CaCO_3_ was added at 0.5% (wt/vol) to the medium to control pH. The cell pellets were harvested by centrifugation (5,000 × *g*, 4°C, 10 min), dissolved in B-PER reagent (Thermo Scientific Pierce), and vortexed for 1 min for cell lysis. The cell lysate was then heat treated at 60°C for 30 min to remove the heat-unstable proteins. Finally, the cell lysate was centrifuged at 12,000 × *g* for 30 min, and the supernatant was used for β-galactosidase assays as previously reported (34).
