# Supplementary material for: A Flexible Binding Site Architecture Provides New Insights into CcpA Global Regulation in Gram-Positive Bacteria
Source: mBio. 2017 Jan 24;8(1):e02004-16. doi: 10.1128/mBio.02004-16 (PMC5263246; doi:10.1128/mBio.02004-16)
Supplement: TABLE S1 [file mbo002173155st1.docx]

**Table S****1. Putative CcpA-binding *cre_var_* sites in the chromosome of *C. acetobutylicum*.**

| Locus tag | Gene | Description | *cre_var_*^a^ | Location^b^ | Position^c^ |
| --- | --- | --- | --- | --- | --- |
| CAP0129^*^ |  | glycogen-binding regulatory subunit of S/T protein phosphatase I | AATGTAAATGTACACCTTTACATT | Prom | -156 |
| CAC0804^*^ | *-* | Pectate lyase related protein, secreted | TGTAAAAAAACAACTAATTTATTTACA | Prom | -172 |
| CAP0162^*^ |  | bifunctional acetaldehyde-CoA/alcohol dehydrogenase | AAACTGCTAAATGTAAATTATACGTTTACATTTAGCAGTTT | Prom | -435 |
|  |  |  | TGTAAAAGTTGCTATTTACA | Prom | -325 |
|  |  |  | TATTGTAAACCTTGTTTTGTTTTGCAGTTTACAATA | Prom | -134 |
| CAC1354^*^ | *-* | Phosphotransferase system IIA component | ATGTAAACGGTATCTGTAATAAAGTGTATTTTTTACAT | Prom | -244 |
| CAC0211 | *bioY* | BioY protein precursor | ATTGTAAACCGATGTTATATTATCGGTTTACAAT | Prom | -51 |
| CAC3456 | *-* | Uncharacterized conserved protein, bioY family | TATTGTAAACCTAAACTATAAATATGGTTTACAATA | Prom | -46 |
| CAC2517^*^ | *nrpE* | Extracellular neutral metalloprotease, NPRE | TGTAAATATTTTTTTTACA | Prom | -415 |
|  |  |  | ATGTAAACGGTTATTTTTACAT | Prom | -146 |
| CAC3229 |  | NADH:flavin oxidoreductase, Old Yellow Enzyme family | AAGGGGATGTAAAACTATTTTTGATAGTTTTACATCCCCTT | Prom | -166 |
| CAC2791^*^ | *-* | MoaA/NirJ family Fe-S oxidoreductase | ATGTAAAATAAATTTGTGGAATAAGGTATTTACAT | Prom | -173 |
| CAC2796^*^ | *-* | MoaA/NirJ family Fe-S oxidoreductase | TGTAAATATACTTTACA | Prom | -49 |
| CAC3649^*^ | *spoVT* | Possible stage V sporulation protein T, transcriptional regulator AbrB homolog | TGTAAAAATATAAGCTTTTACA | Prom | -283 |
| CAC2426^*^ | *-* | hypothetical protein CAC2426 | ATAAGGTACTGTAAATTTCATTTTACAGTACCTTAT | Prom | -192 |
| CAC3214^*^ | *-* | Stage V sporulation protein T, AbrB family transcriptional regulator | ATGTAAAAATATTTTACAT | Prom | -38 |
| CAC2697^*^ | *-* | hypothetical protein CAC2697 | TTGTAAATACTTATTATTTTTGCAAAAAGTTTTACAA | Prom | -163 |
| CAC0952^*^ | *-* | hypothetical protein CAC0952 | TTGTAAATATTTTACAA | Prom | -34 |
| CAC1848^*^ | *cmk* | cytidylate kinase | TGTAAATTGACAGACTTCAAGAAAACTTTTACA | Prom | -191 |
| CAP0058^*^ | *-* | Rare lipoprotein A RLPA releated protein | TGTAAAAATATCGCAACTAGTCAAAATATATAGCACATTATTATTTTACA | Prom | -129 |
| CAP0150^*^ | *spoVD* | Cell division protein FtsI | AATTGTAAAAACATATAAATATATGCTATTATATATTTGGTATATTTTTACAATT | Prom | -69 |
| CAC1768 | *-* | Uncharacterized conserved protein, TraB family | TGTAAAAATGTTAAAGAAAGATGGTTTTACA | ORF | 851 |
| CAC0659 | *-* | Predicted Zn-dependent peptidase | TTGTAAAATATAAAAGGATTTTTTACAA | ORF | 1022 |
| CAC0625^*^ | *-* | Possible periplasmic aspartyl protease | TGTAAATAGATTTACA | ORF | 1421 |
| CAC3087^*^ | *-* | Phosphoenolpyruvate-protein kinase (PTS system enzyme I) | TGTAAAGGATGTTGGAAATAGAGTTTTACA | ORF | 383 |
| CAC2533^*^ | *-* | Protein containing ChW-repeats | TGTAAAGGCGGAATCTTTACA | ORF | 80 |
| CAC3271^*^ | *-* | Transcriptional regulator, AcrR family | TGTAAAGGAAATGATAGAGGGCCATATTTTACA | ORF | 401 |
| CAC3080^*^ | *-* | hypothetical protein CAC3080 | TGTAAATAAAAGAATATATATATGGATTTTTACA | Prom | -62 |
| CAP0168^*^ |  | Alpha-amylase | AATGTAAAGAAATAATTTACATT | Prom | -136 |
| CAC1433^*^ | *-* | hypothetical protein CAC1433 | TGTAAAATTTATTGTTTACA | Prom | -128 |
| CAC0826^*^ | *-* | Endoglucanase family 5 | TGTAAATTTCTATTTTTACA | Prom | -230 |
| CAC0206^*^ | *-* | hypothetical protein CAC0206 | TGTAAAAAATACATAACATAGAATTAACTTACTTTTTACA | Prom | -112 |
| CAC3639^*^ | *-* | CRO repressor-like DNA-binding protein | ATTGTAAAAGAGTATTATTAAGGAATAATGTTTACAAT | Prom | -81 |
| CAC2959^*^ | *galK* | galactokinase | TGTAAAATCTAATAAAAACAAGCTTTACA | Prom | -247 |
| CAC0435^*^ | *-* | hypothetical protein CAC0435 | TGTAAAACTAATAAAAATGCTAATTTATTTTACA | ORF | 39 |
| CAP0047^*^ |  | sulphohydrolase/glycosulfatase, Zn-dependent hydrolase | TGTAAACGTTCCATTTACA | ORF | 284 |
| CAC1286^*^ | *-* | Fe-S oxidoreductase | TGTAAAAGATGTATTTACA | ORF | 359 |
| CAC0910^*^ | *-* | Probably cellulosomal scaffolding protein precursor, secreted; cellulose-binding and cohesin domain | ATGTAAATGTAGCTATGCAATTAAACGGATTTACAT | ORF | 635 |
| CAC0913^*^ | *-* | Possible non-processive endoglucanase family 9, secreted; CelG ortholog; dockerin and cellulose-binding domain | TGTAAATGTAGATTTTACA | ORF | 1724 |
| CAC1217^*^ | *-* | Guanylate kinase (P-loop type) | TGTAAAGGGTATTTTTTTACA | ORF | 335 |
| CAC1812^*^ | *-* | Cell division protein FtsK | TGTAAAGCTTCTTATGATAGATCCTAAGGTAGTTGAATTGAGTGTTTACA | ORF | 1430 |
| CAC1883^*^ | *-* | Phage tail length tape-measure protein | TGTAAAATCTGTTATGTCGCAAATGTCAAATTCATCTTTACA | ORF | 755 |
| CAC3684^*^ | *-* | Polygalacturonase | TGTAAAAAGGGAGCATCTATAAATTATCTTCAAGATAGTACTTTTACA | ORF | 1467 |
| CAC2455^*^ | *-* | hypothetical protein | TGTAAAAATGGTACTGATAGTAAGGATATAACCAAGATTGTTTTTACA | ORF | 669 |
| CAC1128^*^ | *-* | hypothetical protein CAC1128 | TGTAAAAGAAATGAATATATTTTTACA | ORF | 53 |
| CAC2691^*^ | *-* | D-lactate dehydrogenase | TGTAAATGATTATTCTCTTGGCGGTTTACA | ORF | 380 |
| CAC1367^*^ | *cbiQ* | Cobalt permease | TTTGTAAATGTGATTTTTACAAA | ORF | 239 |
| CAC3079 | *-* | Uncharacterized conserved protein, YPPB B.subtilis ortholog | TGTAAAAATCCATATATATATTCTTTTATTTACA | Prom | -145 |
| CAC0485 | *-* | TRNA-processing ribonuclease BN | AGAGATTATGTAAAATTGTAAAGTGGTTTTACATAATCTCT | Prom | -144 |
| CAC2495 | *-* | Predicted transcriptional regulator | TGTAAAGGTAGCTTTACA | Prom | -35 |
| CAC2062 | *-* | TRNA nucleotidyltransferase family enzyme | ATAAAATAAACTGTAAAACTGTTTTAATGGCTTTACAGTTTATTTTAT | Prom | -58 |
| CAC1389 | *-* | Protein containing ChW-repeats and cell-adhesion domain | TGTAAATGAAAAGCAATAAGTGAAAATTGATTTACA | Prom | -52 |
| CAC0951 | *-* | Ferric uptake regulation protein | TGTAAAAAGGCATTATTTAAGTTTTACA | Prom | -121 |
| CAC3333 | *-* | Uncharacterized conserved protein, related to pyruvate formate-lyase activating enzyme | TGTAAATTAGGCTGCTTATTAATAAATACATGTTTTATTTACA | Prom | -168 |
| CAC1251 | *rodA* | Cell division protein, rodA/ftsW/spoVE family | TGTAAATAATGTTTACA | Prom | -109 |
| CAC1532 | *-* | Protein containing ChW-repeats | TGTAAAGTTGAGTATGATTTACA | Prom | -116 |
| CAC2274 | *spoT* | RelA/SpoT protein, (p)ppGpp synthetase/pyrophosphohydrolase | ATTGTAAAAAGTAAACAAAAGTCTAATCTCTTTACAAT | Prom | -124 |
| CAC3273 | *-* | Possible surface protein, responsible for cell interaction; contains cell adhesion domain and ChW-repeats | TATGTAAATCTTTAAAATTAAGCCCTGATGTTAATTAATTACATTTTTTACATA | Prom | -113 |
| CAC2491 | *-* | Histone acetyltransferase HPA2 and related acetyltransferases | TGTAAACTTCCATAATAAGCATATTATATTCTGCGTCTTTTTACA | Prom | -151 |
| CAC2125 | *divIB* | Cell division protein FtsQ | TGTAAATTCAAAGACAGTATAAATAATTTTTTCTTGAATTTAGTTTACA | Prom | -82 |
| CAC2889 | *rho* | termination factor Rho | TGTAAATTATGTATTGAAAATAAAATAAAGTTGCAAATAATTTTTACA | Prom | -75 |
| CAC0453 | *-* | ABC transporter ATP-binding protein | ATGTGTAAAATTTATAAAGTTAAAACACCCGCGTTCTAAAAATTTTACACAT | Prom | -394 |
| CAC1597 | *-* | Zn-finger DNA-binding domain | TGTAAAAAGACCTTTACTGATTTTACA | ORF | 228 |
| CAC0818 | *-* | Diguanylate cyclase/phosphodiesterase domain (GGDEF) containing protein | ATTGTAAAATTAGAAGGTCAATTAGAACTTTACAAT | ORF | 143 |
| CAC2642 | *-* | Predicted endonuclease | TGTAAATTATGATGGAAATTTAATAATAGAAGTTTACA | ORF | 680 |
| CAC2647 | *-* | Diverged arginase family hydrolase | TGTAAACTTACTAATATTGAAAGTTATAATAATGTTTACA | ORF | 492 |
| CAC0045 |  | glycosyl transferase | TGTAAAAGCTGCTGTAGAGCTTTACA | ORF | 281 |
| CAC0771 | *-* | cobalt transport protein CbiM | TGTAAAGCAAATTAAAAATCTTATAGGTTTTACA | ORF | 770 |
| CAC1014 | *-* | IAA-like amino acid hydrolase | TTGTAAATGCGGCTTCAAACCCATTTACAA | ORF | 536 |
| CAC2536 | *-* | Glycosyltransferase | TGTAAAATTTACA | ORF | 788 |
| CAC3224 | *purR* | purine operon repressor | TTGTAAAAAAATTATGTTTGATTTTACAA | ORF | 251 |
| CAC1651 | *-* | Predicted GTPase with uncharacterized domain, ortholog of T.maritima (4980952) | TGTAAAAGGTACAACAACTGATCCTGTTTACA | ORF | 125 |
| CAC1764 | *-* | Predicted glutamine amidotransferase | ATGTAAAAATGAAAATGGCAATCTTTTTACAT | ORF | 30 |
| CAC1728 | *-* | Serine/threonine protein kinase, Pkn2 family (YLOP B.subtilis ortholog) | CTGTAAAAGTTCTTAAACATGAATTTACAG | ORF | 110 |
|  |  |  | TGTAAATGATGATGAAGAGTTTACA | ORF | 818 |
| CAC2187 | *spsE* | Sialic acid synthase | TTGTAAAAGATATAAAAAAAGGCGAGACATTTACAA | ORF | 890 |
| CAC1844 | *-* | hypothetical protein CAC1844 | AAAATGTAAAAAACAATATTTACATTTT | ORF | 119 |
| CAC3398 | *-* | Uncharacterized conserved protein, YbhB family | ATGTAAAGCTTAATCTTAAAGATGGATTTTACAT | ORF | 392 |
| CAC2622 | *comE* | ComE-like protein, Metallo beta-lactamase superfamily hydrolase | TGTAAAAGTTACATATGGACAAAACAAATTTTTGTTTACA | ORF | 542 |
| CAC2724 | *-* | hypothetical protein CAC2724 | TGTAAACTTTGTTTTACA | ORF | 36 |
| CAC1726 | *-* | Predicted Fe-S-cluster redox enzyme, YLON B.subtilis ortholog | CTGTAAAAAATACTCTGATTTTACAG | ORF | 717 |
| CAC2937 | *-* | Ketopantoate reductase PanE/ApbA | TGTAAAAAACCTTTACA | ORF | 227 |
| CAC3006 | *-* | Zn-dependent peptidase, insulinase family | TGTAAAAAGCGGAGCTAGACTACTATATTTACA | ORF | 98 |
| CAC2432 | *-* | Predicted permease | TATTGTAAATCAAGCAACTAACTTTTACAATA | ORF | 293 |
| CAC0903 | *-* | Sensory transduction histidine kinase | TGTAAAGTTTACA | ORF | 1679 |
| CAC1688 | *-* | Penicillin-binding protein | TGTAAATGATAAAGTAAAAACAAATCAAATTTACA | ORF | 1253 |
| CAC1838 | *-* | Predicted Fe-S oxidoreductase, YMCB B.subtilis ortholog | TGTAAAAGAGGTAGAGTACGATTCAGCATTTACA | ORF | 974 |
| CAC2325 | *-* | Possible cell wall hydrolase containing N-acetylglucosaminidase domain and ChW-repeats | TGTAAATGGTACAATTGTTTACA | ORF | 1964 |
| CAC3390 | *-* | Response regulator (CheY-like receiver domain containing) | TGTAAAGGTGGAGGATTCTGTAGAGGAGCTTTACA | ORF | 1052 |
| CAC3402 | *-* | Dipeptidyl aminopeptidase/acylaminoacyl-peptidase related protein | TGTAAAAAGGAATACATGTTTTTTACA | ORF | 1083 |
| CAC2755 | *-* | MDR-type ABC transporter | TGTAAAAAAGAATATTTTGTACATAGTTTTAATAGCCTTTTTTACA | ORF | 161 |
| CAC3414 | *-* | ABC-type multidrug/protein/lipid transport system, ATPase component | TGTAAAAGCAGAGAAGATTATTATACTAAATGCTCCACTAATGCAGTTTACA | ORF | 689 |
| CAC2107 | *-* | Contains cell adhesion domain | TGTAAAAAATCAATTTGTAACACTTGCTTATGAAAAATCAGATTTACA | ORF | 416 |
| CAC3343 | *-* | Biotin synthase related domain containing protein | GTGTAAATAGAATTTCAAACGATACTCCTCGCGCTTCATTTACAC | ORF | 212 |
| CAC0239 | *-* | Histidine kinase-like ATPase | TGTAAATATAGACATAAATGATAAGTATATAAGGCTTTACA | ORF | 884 |
| CAC1387 | *-* | Membrane associated chemotaxis sensory transducer protein | TGTAAACAAAGGCGATTATGATAAGGCTAATGAATATTTTACA | ORF | 407 |
| CAC2623 | *-* | Possible sensorory transduction protein | TGTAAATAATAATATAAGTCAATTTATAAATTTATTTAAAATTAGTTTTACA | ORF | 1430 |
| CAC2431 | *-* | Lipoteichoic acid synthase LtaS Type IVa | TGTAAAAGATGGTGATAGTACCAATGTTACATTTAGAAATGGATCGTTTACA | ORF | 1682 |
| CAC0916 | *-* | Possible non-processive endoglucanase family 9, secreted | TGTAAAAAAAGGACAAGATAAATACATGTTAAATATATTAAAGCATTTTACA | ORF | 335 |
| CAC1949 | *-* | Possible TPR-repeat contaning protein | TTTGTAAATTATGCTATTAGGAATAAAAAGAGCAAGGAAATTTTTACAAA | ORF | 1037 |
| CAC1595 | *-* | Integrase/recombinase, XerC/XerD family | TGTAAAAAAGAAGCAGATGAGCATGGACTCGTATTTATTTTACA | ORF | 263 |
| CAC3376 | *-* | Possible pectin degradation protein (sugar phosphate isomerase family) | TGTAAATGTCTTAAAAAGGGAGTGCTCTTGGATGTGTTTACA | ORF | 264 |
| CAC0305 | *motB* | chemotaxis protein MotB | TGTAAACCAAAACGGTATGCAGGGCAGTGTTAGCACAAATTTACA | ORF | 323 |

*The genes that showed over 2-fold transcriptional change in at least one phase after *ccpA* inactivation.

^a^ The bases in red represent the 6-bp core sequences within the two inverted repeats.

^b^ Prom:promoter region; ORF:open reading frame.

^c^ The position of the leftmost base"T" within the left 6-bp core sequence (TGTAAA) relative to the respective translation start point of the associated gene.
