# Supplementary figures and images for: A Flexible Binding Site Architecture Provides New Insights into CcpA Global Regulation in Gram-Positive Bacteria
Source: mBio. 2017 Jan 24;8(1):e02004-16. doi: 10.1128/mBio.02004-16 (PMC5263246; doi:10.1128/mBio.02004-16)

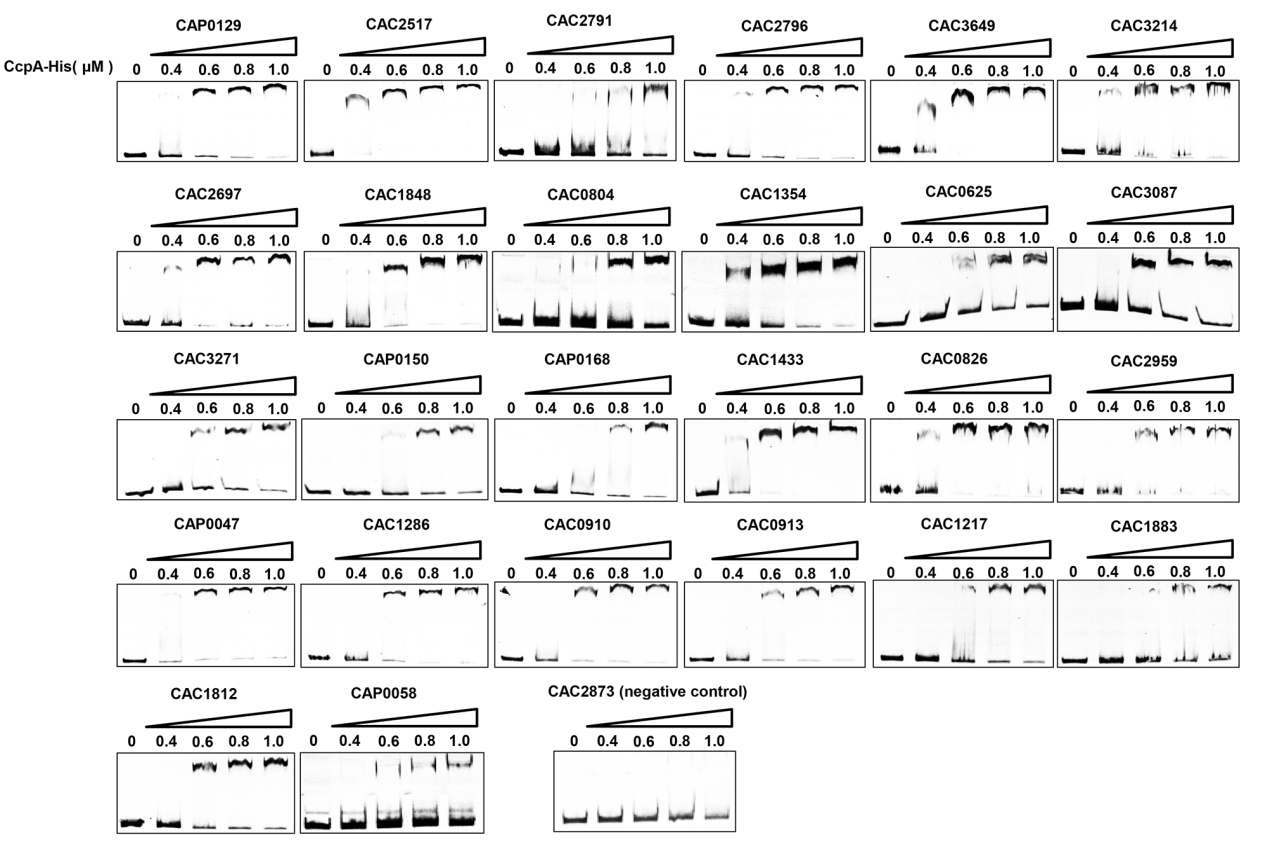

Supplement: FIG S1 [file mbo002173155sf1.docx]

**
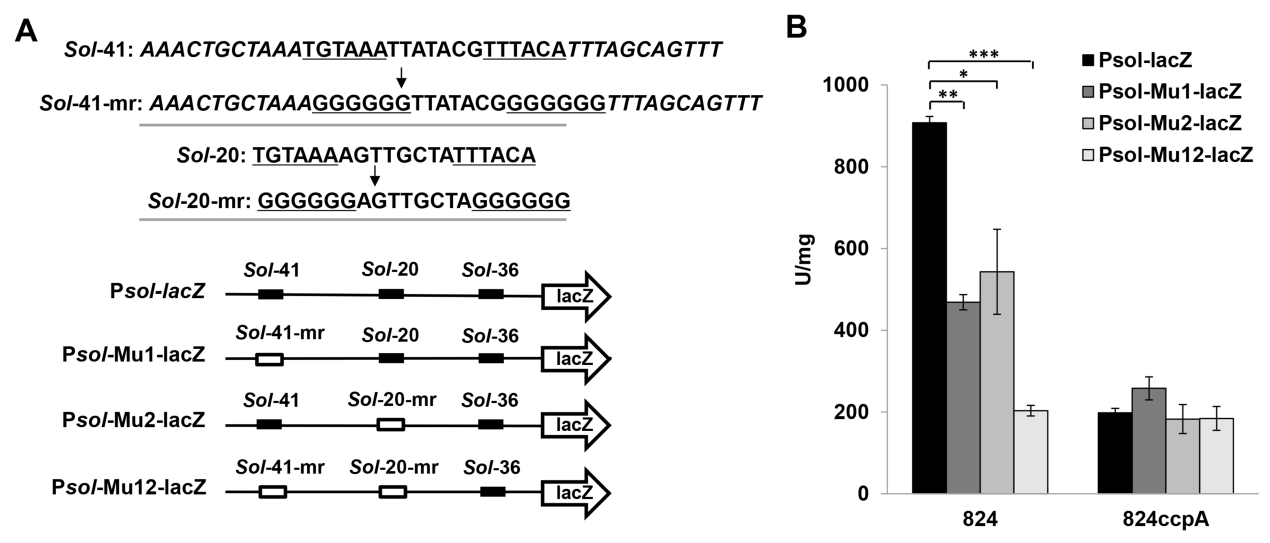
**

Supplement: FIG S2 [file mbo002173155sf2.docx]
