# Supplementary material for: A Flexible Binding Site Architecture Provides New Insights into CcpA Global Regulation in Gram-Positive Bacteria
Source: mBio. 2017 Jan 24;8(1):e02004-16. doi: 10.1128/mBio.02004-16 (PMC5263246; doi:10.1128/mBio.02004-16)
Supplement: TABLE S2 [file mbo002173155st2.docx]

**Table S2. CcpA –binding *cre_var_* sites identified in *C. acetobutylicum*.**

| Locus tag | Gene | Description | *cre_var_*^a^ | Location^b^ | Position^c^ |
| --- | --- | --- | --- | --- | --- |
| CAP0129 | *-* | glycogen-binding regulatory subunit of S/T protein phosphatase I | AATGTAAATGTACACCTTTACATT | Prom | -156 |
| CAC0804 | *-* | Pectate lyase related protein, secreted | TGTAAAAAAACAACTAATTTATTTACA | Prom | -172 |
| CAP0162 | *adhE1* | bifunctional acetaldehyde-CoA/alcohol dehydrogenase | AAACTGCTAAATGTAAATTATACGTTTACATTTAGCAGTTT | Prom | -435 |
|  | *adhE1* | bifunctional acetaldehyde-CoA/alcohol dehydrogenase | TGTAAAAGTTGCTATTTACA | Prom | -325 |
|  | *adhE1* | bifunctional acetaldehyde-CoA/alcohol dehydrogenase | TATTGTAAACCTTGTTTTGTTTTGCAGTTTACAATA | Prom | -134 |
| CAC1354 | *-* | Phosphotransferase system IIA component | ATGTAAACGGTATCTGTAATAAAGTGTATTTTTTACAT | Prom | -244 |
| CAC2517 | *nrpE* | Extracellular neutral metalloprotease, NPRE | TGTAAATATTTTTTTTACA | Prom | -415 |
|  | *nrpE* | Extracellular neutral metalloprotease, NPRE | ATGTAAACGGTTATTTTTACAT | Prom | -146 |
| CAC2791 | *-* | MoaA/NirJ family Fe-S oxidoreductase | ATGTAAAATAAATTTGTGGAATAAGGTATTTACAT | Prom | -173 |
| CAC2796 | *-* | MoaA/NirJ family Fe-S oxidoreductase | TGTAAATATACTTTACA | Prom | -49 |
| CAC3649 | *spoVT* | Possible stage V sporulation protein T | TGTAAAAATATAAGCTTTTACA | Prom | -283 |
| CAC3214 | *-* | Stage V sporulation protein T | ATGTAAAAATATTTTACAT | Prom | -38 |
| CAC2697 | *-* | hypothetical protein CAC2697 | TTGTAAATACTTATTATTTTTGCAAAAAGTTTTACAA | Prom | -163 |
| CAC1848 | *cmk* | cytidylate kinase | TGTAAATTGACAGACTTCAAGAAAACTTTTACA | Prom | -191 |
| CAP0058 | *-* | Rare lipoprotein A RLPA releated protein | TGTAAAAATATCGCAACTAGTCAAAATATATAGCACATTATTATTTTACA | Prom | -129 |
| CAP0150 | *spoVD* | Cell division protein FtsI | AATTGTAAAAACATATAAATATATGCTATTATATATTTGGTATATTTTTACAATT | Prom | -69 |
| CAC0625 | *-* | Possible periplasmic aspartyl protease | TGTAAATAGATTTACA | ORF | 1421 |
| CAC3087 | *-* | Phosphoenolpyruvate-protein kinase (PTS system enzyme I) | TGTAAAGGATGTTGGAAATAGAGTTTTACA | ORF | 383 |
| CAC3271 | *-* | Transcriptional regulator, AcrR family | TGTAAAGGAAATGATAGAGGGCCATATTTTACA | ORF | 401 |
| CAP0168 | *-* | Alpha-amylase | AATGTAAAGAAATAATTTACATT | Prom | -136 |
| CAC1433 | *-* | hypothetical protein CAC1433 | TGTAAAATTTATTGTTTACA | Prom | -128 |
| CAC0826 | *-* | Endoglucanase family 5 | TGTAAATTTCTATTTTTACA | Prom | -230 |
| CAC2959 | *galK* | galactokinase | TGTAAAATCTAATAAAAACAAGCTTTACA | Prom | -247 |
| CAP0047 |  | sulphohydrolase/glycosulfatase, Zn-dependent hydrolase | TGTAAACGTTCCATTTACA | ORF | 284 |
| CAC1286 | *-* | Fe-S oxidoreductase | TGTAAAAGATGTATTTACA | ORF | 359 |
| CAC0910 | *-* | Probably cellulosomal scaffolding protein precursor, secreted | ATGTAAATGTAGCTATGCAATTAAACGGATTTACAT | ORF | 635 |
| CAC0913 | *-* | Possible non-processive endoglucanase family 9, secreted | TGTAAATGTAGATTTTACA | ORF | 1724 |
| CAC1217 | *-* | Guanylate kinase (P-loop type) | TGTAAAGGGTATTTTTTTACA | ORF | 335 |
| CAC1883 | *-* | Phage tail length tape-measure protein | TGTAAAATCTGTTATGTCGCAAATGTCAAATTCATCTTTACA | ORF | 755 |
| CAC1812 | *-* | Cell division protein FtsK | TGTAAAGCTTCTTATGATAGATCCTAAGGTAGTTGAATTGAGTGTTTACA | ORF | 1430 |

^a^ The bases in red represent the 6-bp core sequences within the two inverted repeats.

^b^ Prom:promoter region; ORF:open reading frame.

^c^ The position of the leftmost base"T" within the left 6-bp core sequence (TGTAAA) relative to the respective translation start point of the associated gene.
