# Supplementary material for: A Flexible Binding Site Architecture Provides New Insights into CcpA Global Regulation in Gram-Positive Bacteria
Source: mBio. 2017 Jan 24;8(1):e02004-16. doi: 10.1128/mBio.02004-16 (PMC5263246; doi:10.1128/mBio.02004-16)
Supplement: TABLE S3 [file mbo002173155st3.docx]

**Table S3.** **Putative *cre_var_* sites in class Clostridia and class Bacilli.**

| Locus tag | *cre_var_* | Length of intervening spacer | Position^a^ | Location^b^ |
| --- | --- | --- | --- | --- |
| **The *cre_var_* sites in class Clostridia** |  |  |  |  |
| **1. *Clostridium beijerinckii* NCIMB 8052** |  |  |  |  |
| Cbei_2444 | TGTAAA-(0)-TTTACA | 0 | -51 | Prom |
| Cbei_4245 | TGTAAA-(12)-TTTACA | 12 | -182 | Prom |
| Cbei_2155 | TGTAAA-(3)-TTTACA | 3 | -82 | Prom |
| Cbei_2894 | TGTAAA-(14)-TTTACA | 14 | -124 | Prom |
| Cbei_4923 | TGTAAA-(9)-TTTACA | 9 | -48 | Prom |
| Cbei_2674 | TGTAAA-(12)-TTTACA | 12 | -52 | Prom |
| Cbei_3360 | TGTAAA-(18)-TTTACA | 18 | -98 | Prom |
| Cbei_3030 | TGTAAA-(10)-TTTACA | 10 | -187 | Prom |
| Cbei_2185 | TGTAAA-(11)-TTTACA | 11 | -183 | Prom |
| Cbei_0866 | TGTAAA-(15)-TTTACA | 15 | -174 | Prom |
| Cbei_3361 | TGTAAA-(18)-TTTACA | 18 | -61 | Prom |
| Cbei_3766 | TGTAAA-(2)-TTTACA | 2 | -48 | Prom |
| Cbei_3084 | TGTAAA-(5)-TTTACA | 5 | -32 | Prom |
| Cbei_4977 | TGTAAA-(4)-TTTACA | 4 | -162 | Prom |
| Cbei_4067 | TGTAAA-(19)-TTTACA | 19 | -205 | Prom |
| Cbei_0487 | TGTAAA-(22)-TTTACA | 22 | -234 | Prom |
| Cbei_0879 | TGTAAA-(26)-TTTACA | 26 | -111 | Prom |
| Cbei_0941 | TGTAAA-(23)-TTTACA | 23 | -153 | Prom |
| Cbei_4861 | TGTAAA-(19)-TTTACA | 19 | -85 | Prom |
| Cbei_3759 | TGTAAA-(28)-TTTACA | 28 | -55 | Prom |
| Cbei_3866 | TGTAAA-(24)-TTTACA | 24 | -93 | Prom |
| Cbei_1314 | TGTAAA-(38)-TTTACA | 38 | -50 | Prom |
| Cbei_1392 | TGTAAA-(39)-TTTACA | 39 | -134 | Prom |
| Cbei_1391 | TGTAAA-(27)-TTTACA | 27 | -113 | Prom |
| Cbei_4577 | TGTAAA-(34)-TTTACA | 34 | -236 | Prom |
| Cbei_2764 | TGTAAA-(27)-TTTACA | 27 | -63 | Prom |
| Cbei_0575 | TGTAAA-(31)-TTTACA | 31 | -247 | Prom |
| Cbei_4686 | TGTAAA-(34)-TTTACA | 34 | -238 | Prom |
| Cbei_2391 | TGTAAA-(21)-TTTACA | 21 | -114 | Prom |
| Cbei_0576 | TGTAAA-(31)-TTTACA | 31 | -356 | Prom |
| Cbei_2896 | TGTAAA-(14)-TTTACA | 14 | -378 | Prom |
| Cbei_1928 | TGTAAA-(35)-TTTACA | 35 | -411 | Prom |
| Cbei_0880 | TGTAAA-(26)-TTTACA | 26 | -201 | Prom |
| Cbei_2306 | TGTAAA-(3)-TTTACA | 3 | -381 | Prom |
| Cbei_1315 | TGTAAA-(38)-TTTACA | 38 | -270 | Prom |
| Cbei_0871 | TGTAAA-(14)-TTTACA | 14 | -373 | Prom |
| Cbei_3865 | TGTAAA-(24)-TTTACA | 24 | -213 | Prom |
| Cbei_2645 | TGTAAA-(32)-TTTACA | 32 | -384 | Prom |
| Cbei_0310 | TGTAAA-(27)-TTTACA | 27 | -320 | Prom |
| Cbei_3767 | TGTAAA-(2)-TTTACA | 2 | -380 | Prom |
| Cbei_2156 | TGTAAA-(3)-TTTACA | 3 | -483 | Prom |
| Cbei_2196 | TGTAAA-(12)-TTTACA | 12 | 647 | ORF |
| Cbei_1974 | TGTAAA-(16)-TTTACA | 16 | 1205 | ORF |
| Cbei_1712 | TGTAAA-(19)-TTTACA | 19 | 323 | ORF |
| Cbei_2328 | TGTAAA-(4)-TTTACA | 4 | 299 | ORF |
| Cbei_2702 | TGTAAA-(34)-TTTACA | 34 | 254 | ORF |
| Cbei_4336 | TGTAAA-(16)-TTTACA | 16 | 1136 | ORF |
| Cbei_4432 | TGTAAA-(7)-TTTACA | 7 | 26 | ORF |
| Cbei_1936 | TGTAAA-(13)-TTTACA | 13 | 353 | ORF |
| Cbei_4435 | TGTAAA-(0)-TTTACA | 0 | 134 | ORF |
| Cbei_3184 | TGTAAA-(5)-TTTACA | 5 | 236 | ORF |
| Cbei_4687 | TGTAAA-(6)-TTTACA | 6 | 980 | ORF |
| Cbei_1088 | TGTAAA-(28)-TTTACA | 28 | 128 | ORF |
| Cbei_2075 | TGTAAA-(40)-TTTACA | 40 | 53 | ORF |
| Cbei_1827 | TGTAAA-(22)-TTTACA | 22 | 335 | ORF |
| Cbei_3207 | TGTAAA-(10)-TTTACA | 10 | 362 | ORF |
| Cbei_4973 | TGTAAA-(33)-TTTACA | 33 | 914 | ORF |
| Cbei_0996 | TGTAAA-(31)-TTTACA | 31 | 749 | ORF |
| Cbei_4373 | TGTAAA-(19)-TTTACA | 19 | 164 | ORF |
| Cbei_3403 | TGTAAA-(32)-TTTACA | 32 | 560 | ORF |
| Cbei_2047 | TGTAAA-(17)-TTTACA | 17 | 557 | ORF |
| Cbei_2769 | TGTAAA-(31)-TTTACA | 31 | 833 | ORF |
| Cbei_4211 | TGTAAA-(9)-TTTACA | 9 | 686 | ORF |
| Cbei_0455 | TGTAAA-(9)-TTTACA | 9 | 1034 | ORF |
| Cbei_3488 | TGTAAA-(27)-TTTACA | 27 | 345 | ORF |
| Cbei_1229 | TGTAAA-(8)-TTTACA | 8 | 1178 | ORF |
| Cbei_1464 | TGTAAA-(36)-TTTACA | 36 | 77 | ORF |
| Cbei_2518 | TGTAAA-(31)-TTTACA | 31 | 207 | ORF |
| Cbei_3176 | TGTAAA-(8)-TTTACA | 8 | 464 | ORF |
| Cbei_3383 | TGTAAA-(22)-TTTACA | 22 | 1175 | ORF |
| Cbei_3765 | TGTAAA-(0)-TTTACA | 0 | 1895 | ORF |
| Cbei_2589 | TGTAAA-(14)-TTTACA | 14 | 795 | ORF |
| Cbei_0968 | TGTAAA-(20)-TTTACA | 20 | 485 | ORF |
| Cbei_4791 | TGTAAA-(30)-TTTACA | 30 | 956 | ORF |
| Cbei_3773 | TGTAAA-(19)-TTTACA | 19 | 872 | ORF |
| Cbei_1152 | TGTAAA-(11)-TTTACA | 11 | 726 | ORF |
| Cbei_4247 | TGTAAA-(22)-TTTACA | 22 | 212 | ORF |
| Cbei_4551 | TGTAAA-(19)-TTTACA | 19 | 266 | ORF |
| Cbei_0798 | TGTAAA-(24)-TTTACA | 24 | 818 | ORF |
| Cbei_1418 | TGTAAA-(32)-TTTACA | 32 | 65 | ORF |
| Cbei_5092 | TGTAAA-(27)-TTTACA | 27 | 425 | ORF |
| Cbei_2542 | TGTAAA-(16)-TTTACA | 16 | 137 | ORF |
| Cbei_0871 | TGTAAA-(7)-TTTACA | 7 | 680 | ORF |
| Cbei_4420 | TGTAAA-(30)-TTTACA | 30 | 59 | ORF |
| Cbei_3667 | TGTAAA-(18)-TTTACA | 18 | 332 | ORF |
| Cbei_0914 | TGTAAA-(40)-TTTACA | 40 | 293 | ORF |
| Cbei_1357 | TGTAAA-(20)-TTTACA | 20 | 488 | ORF |
| Cbei_1426 | TGTAAA-(24)-TTTACA | 24 | 537 | ORF |
| Cbei_1662 | TGTAAA-(22)-TTTACA | 22 | 920 | ORF |
| Cbei_1701 | TGTAAA-(39)-TTTACA | 39 | 158 | ORF |
| 1. ***Clostridium botulinum* A str. ATCC 3502** |  |  |  |  |
| CBO2943 | TGTAAA-(0)-TTTACA | 0 | -482 | Prom |
| CBO1613 | TGTAAA-(2)-TTTACA | 2 | -479 | Prom |
| CBO0695 | TGTAAA-(3)-TTTACA | 3 | -425 | Prom |
| CBO3330 | TGTAAA-(17)-TTTACA | 17 | -415 | Prom |
| CBO1166 | TGTAAA-(20)-TTTACA | 20 | -401 | Prom |
| CBO0526 | TGTAAA-(16)-TTTACA | 16 | -340 | Prom |
| CBO0479A | TGTAAA-(8)-TTTACA | 8 | -311 | Prom |
| CBO2243 | TGTAAA-(30)-TTTACA | 30 | -306 | Prom |
| CBO2227 | TGTAAA-(20)-TTTACA | 20 | -281 | Prom |
| CBO0527 | TGTAAA-(16)-TTTACA | 16 | -212 | Prom |
| CBO2821 | TGTAAA-(20)-TTTACA | 20 | -210 | Prom |
| CBO0804 | TGTAAA-(36)-TTTACA | 36 | -161 | Prom |
| CBO3490 | TGTAAA-(30)-TTTACA | 30 | -161 | Prom |
| CBO0696 | TGTAAA-(3)-TTTACA | 3 | -153 | Prom |
| CBO1614 | TGTAAA-(2)-TTTACA | 2 | -129 | Prom |
| CBO3053 | TGTAAA-(30)-TTTACA | 30 | -120 | Prom |
| CBO3329 | TGTAAA-(17)-TTTACA | 17 | -118 | Prom |
| CBO0284 | TGTAAA-(31)-TTTACA | 31 | -116 | Prom |
| CBO2321 | TGTAAA-(38)-TTTACA | 38 | -105 | Prom |
| CBO1547 | TGTAAA-(33)-TTTACA | 33 | -104 | Prom |
| CBO2213 | TGTAAA-(25)-TTTACA | 25 | -101 | Prom |
| CBO3052 | TGTAAA-(30)-TTTACA | 30 | -93 | Prom |
| CBO2214 | TGTAAA-(25)-TTTACA | 25 | -79 | Prom |
| CBO0352 | TGTAAA-(10)-TTTACA | 10 | -73 | Prom |
| CBO2320 | TGTAAA-(38)-TTTACA | 38 | -73 | Prom |
| CBO0976 | TGTAAA-(39)-TTTACA | 39 | -69 | Prom |
| CBO1886 | TGTAAA-(24)-TTTACA | 24 | -67 | Prom |
| CBO3524 | TGTAAA-(22)-TTTACA | 22 | -65 | Prom |
| CBO1548 | TGTAAA-(33)-TTTACA | 33 | -59 | Prom |
| CBO2288 | TGTAAA-(27)-TTTACA | 27 | -53 | Prom |
| CBO3435 | TGTAAA-(25)-TTTACA | 25 | -50 | Prom |
| CBO1539 | TGTAAA-(37)-TTTACA | 37 | 11 | ORF |
| CBO0207 | TGTAAA-(37)-TTTACA | 37 | 17 | ORF |
| CBO2398 | TGTAAA-(12)-TTTACA | 12 | 20 | ORF |
| CBO2079 | TGTAAA-(34)-TTTACA | 34 | 35 | ORF |
| CBO1713 | TGTAAA-(26)-TTTACA | 26 | 36 | ORF |
| CBO3328 | TGTAAA-(11)-TTTACA | 11 | 39 | ORF |
| CBO1774 | TGTAAA-(7)-TTTACA | 7 | 56 | ORF |
| CBO1998 | TGTAAA-(35)-TTTACA | 35 | 59 | ORF |
| CBO2217 | TGTAAA-(36)-TTTACA | 36 | 77 | ORF |
| CBO2509 | TGTAAA-(7)-TTTACA | 7 | 80 | ORF |
| CBO3521 | TGTAAA-(19)-TTTACA | 19 | 84 | ORF |
| CBO2422 | TGTAAA-(22)-TTTACA | 22 | 93 | ORF |
| CBO1921 | TGTAAA-(3)-TTTACA | 3 | 116 | ORF |
| CBO1060 | TGTAAA-(14)-TTTACA | 14 | 147 | ORF |
| CBO1987 | TGTAAA-(7)-TTTACA | 7 | 152 | ORF |
| CBO0539 | TGTAAA-(23)-TTTACA | 23 | 233 | ORF |
| CBO3099 | TGTAAA-(40)-TTTACA | 40 | 242 | ORF |
| CBO3454 | TGTAAA-(36)-TTTACA | 36 | 263 | ORF |
| CBO0273 | TGTAAA-(34)-TTTACA | 34 | 266 | ORF |
| CBO0991 | TGTAAA-(27)-TTTACA | 27 | 272 | ORF |
| CBO0024 | TGTAAA-(9)-TTTACA | 9 | 275 | ORF |
| CBO3022 | TGTAAA-(2)-TTTACA | 2 | 275 | ORF |
| CBO2176 | TGTAAA-(37)-TTTACA | 37 | 332 | ORF |
| CBO0526 | TGTAAA-(11)-TTTACA | 11 | 342 | ORF |
| CBO1024 | TGTAAA-(3)-TTTACA | 3 | 362 | ORF |
| CBO1580 | TGTAAA-(4)-TTTACA | 4 | 383 | ORF |
| CBO3517 | TGTAAA-(9)-TTTACA | 9 | 420 | ORF |
| CBO2278 | TGTAAA-(16)-TTTACA | 16 | 431 | ORF |
| CBO0674 | TGTAAA-(25)-TTTACA | 25 | 453 | ORF |
| CBO3157 | TGTAAA-(2)-TTTACA | 2 | 477 | ORF |
| CBO0784 | TGTAAA-(39)-TTTACA | 39 | 509 | ORF |
| CBO3483 | TGTAAA-(14)-TTTACA | 14 | 536 | ORF |
| CBO2721 | TGTAAA-(11)-TTTACA | 11 | 566 | ORF |
| CBO3024 | TGTAAA-(30)-TTTACA | 30 | 567 | ORF |
| CBO1853 | TGTAAA-(16)-TTTACA | 16 | 599 | ORF |
| CBO1181 | TGTAAA-(21)-TTTACA | 21 | 611 | ORF |
| CBO3233 | TGTAAA-(40)-TTTACA | 40 | 666 | ORF |
| CBO3268 | TGTAAA-(0)-TTTACA | 0 | 764 | ORF |
| CBO0190 | TGTAAA-(24)-TTTACA | 24 | 773 | ORF |
| CBO0028 | TGTAAA-(9)-TTTACA | 9 | 776 | ORF |
| CBO2703 | TGTAAA-(13)-TTTACA | 13 | 776 | ORF |
| CBO3501 | TGTAAA-(34)-TTTACA | 34 | 776 | ORF |
| CBO1802 | TGTAAA-(12)-TTTACA | 12 | 782 | ORF |
| CBO2072 | TGTAAA-(6)-TTTACA | 6 | 812 | ORF |
| CBO2162 | TGTAAA-(16)-TTTACA | 16 | 851 | ORF |
| CBO2415 | TGTAAA-(18)-TTTACA | 18 | 916 | ORF |
| CBO1802 | TGTAAA-(25)-TTTACA | 25 | 947 | ORF |
| CBO0016 | TGTAAA-(12)-TTTACA | 12 | 972 | ORF |
| CBO2519 | TGTAAA-(3)-TTTACA | 3 | 1010 | ORF |
| CBO2068 | TGTAAA-(18)-TTTACA | 18 | 1025 | ORF |
| CBO2972 | TGTAAA-(16)-TTTACA | 16 | 1100 | ORF |
| CBO0801 | TGTAAA-(22)-TTTACA | 22 | 1199 | ORF |
| CBO2595 | TGTAAA-(38)-TTTACA | 38 | 1209 | ORF |
| CBO2708 | TGTAAA-(16)-TTTACA | 16 | 1265 | ORF |
| CBO2990 | TGTAAA-(28)-TTTACA | 28 | 1415 | ORF |
| CBO0028 | TGTAAA-(6)-TTTACA | 6 | 1530 | ORF |
| CBO1456 | TGTAAA-(20)-TTTACA | 20 | 1940 | ORF |
| 1. ***Clostridium butyricum* 5521** |  |  |  |  |
| CBY_3231 | TGTAAA-(19)-TTTACA | 19 | -461 | Prom |
| CBY_0566 | TGTAAA-(19)-TTTACA | 19 | -448 | Prom |
| CBY_2903 | TGTAAA-(32)-TTTACA | 32 | -448 | Prom |
| CBY_3894 | TGTAAA-(12)-TTTACA | 12 | -373 | Prom |
| CBY_1901 | TGTAAA-(10)-TTTACA | 10 | -368 | Prom |
| CBY_0874 | TGTAAA-(40)-TTTACA | 40 | -364 | Prom |
| CBY_3552 | TGTAAA-(25)-TTTACA | 25 | -347 | Prom |
| CBY_3041 | TGTAAA-(11)-TTTACA | 11 | -334 | Prom |
| CBY_0567 | TGTAAA-(19)-TTTACA | 19 | -297 | Prom |
| CBY_3341 | TGTAAA-(36)-TTTACA | 36 | -275 | Prom |
| CBY_3526 | TGTAAA-(25)-TTTACA | 25 | -269 | Prom |
| CBY_0610 | TGTAAA-(1)-TTTACA | 1 | -251 | Prom |
| CBY_1220 | TGTAAA-(23)-TTTACA | 23 | -237 | Prom |
| CBY_3757 | TGTAAA-(2)-TTTACA | 2 | -221 | Prom |
| CBY_1935 | TGTAAA-(23)-TTTACA | 23 | -215 | Prom |
| CBY_3016 | TGTAAA-(27)-TTTACA | 27 | -210 | Prom |
| CBY_0873 | TGTAAA-(40)-TTTACA | 40 | -199 | Prom |
| CBY_0957 | TGTAAA-(8)-TTTACA | 8 | -188 | Prom |
| CBY_0233 | TGTAAA-(34)-TTTACA | 34 | -165 | Prom |
| CBY_3992 | TGTAAA-(17)-TTTACA | 17 | -161 | Prom |
| CBY_3215 | TGTAAA-(21)-TTTACA | 21 | -158 | Prom |
| CBY_0367 | TGTAAA-(13)-TTTACA | 13 | -150 | Prom |
| CBY_2789 | TGTAAA-(21)-TTTACA | 21 | -145 | Prom |
| CBY_1606 | TGTAAA-(17)-TTTACA | 17 | -142 | Prom |
| CBY_1900 | TGTAAA-(10)-TTTACA | 10 | -140 | Prom |
| CBY_3342 | TGTAAA-(36)-TTTACA | 36 | -139 | Prom |
| CBY_1697 | TGTAAA-(33)-TTTACA | 33 | -138 | Prom |
| CBY_0005 | TGTAAA-(29)-TTTACA | 29 | -126 | Prom |
| CBY_0457 | TGTAAA-(15)-TTTACA | 15 | -126 | Prom |
| CBY_4189 | TGTAAA-(12)-TTTACA | 12 | -114 | Prom |
| CBY_1219 | TGTAAA-(23)-TTTACA | 23 | -108 | Prom |
| CBY_0960 | TGTAAA-(33)-TTTACA | 33 | -107 | Prom |
| CBY_0006 | TGTAAA-(29)-TTTACA | 29 | -103 | Prom |
| CBY_2533 | TGTAAA-(15)-TTTACA | 15 | -99 | Prom |
| CBY_2925 | TGTAAA-(20)-TTTACA | 20 | -89 | Prom |
| CBY_4209 | TGTAAA-(6)-TTTACA | 6 | -85 | Prom |
| CBY_4190 | TGTAAA-(12)-TTTACA | 12 | -80 | Prom |
| CBY_1435 | TGTAAA-(20)-TTTACA | 20 | -67 | Prom |
| CBY_3551 | TGTAAA-(25)-TTTACA | 25 | -63 | Prom |
| CBY_0835 | TGTAAA-(31)-TTTACA | 31 | -20 | Prom |
| CBY_1651 | TGTAAA-(6)-TTTACA | 6 | -6 | Prom |
| CBY_1995 | TGTAAA-(6)-TTTACA | 6 | 14 | ORF |
| CBY_1014 | TGTAAA-(21)-TTTACA | 21 | 21 | ORF |
| CBY_3897 | TGTAAA-(21)-TTTACA | 21 | 21 | ORF |
| CBY_0332 | TGTAAA-(32)-TTTACA | 32 | 41 | ORF |
| CBY_2606 | TGTAAA-(4)-TTTACA | 4 | 65 | ORF |
| CBY_2472 | TGTAAA-(36)-TTTACA | 36 | 77 | ORF |
| CBY_1743 | TGTAAA-(19)-TTTACA | 19 | 83 | ORF |
| CBY_1908 | TGTAAA-(5)-TTTACA | 5 | 89 | ORF |
| CBY_0578 | TGTAAA-(19)-TTTACA | 19 | 92 | ORF |
| CBY_0189 | TGTAAA-(13)-TTTACA | 13 | 119 | ORF |
| CBY_3574 | TGTAAA-(16)-TTTACA | 16 | 128 | ORF |
| CBY_0208 | TGTAAA-(10)-TTTACA | 10 | 129 | ORF |
| CBY_3269 | TGTAAA-(7)-TTTACA | 7 | 131 | ORF |
| CBY_3252 | TGTAAA-(33)-TTTACA | 33 | 149 | ORF |
| CBY_1148 | TGTAAA-(34)-TTTACA | 34 | 171 | ORF |
| CBY_0681 | TGTAAA-(38)-TTTACA | 38 | 195 | ORF |
| CBY_1764 | TGTAAA-(38)-TTTACA | 38 | 201 | ORF |
| CBY_3527 | TGTAAA-(33)-TTTACA | 33 | 207 | ORF |
| CBY_0433 | TGTAAA-(35)-TTTACA | 35 | 254 | ORF |
| CBY_3522 | TGTAAA-(3)-TTTACA | 3 | 284 | ORF |
| CBY_3595 | TGTAAA-(15)-TTTACA | 15 | 312 | ORF |
| CBY_1225 | TGTAAA-(40)-TTTACA | 40 | 353 | ORF |
| CBY_3917 | TGTAAA-(37)-TTTACA | 37 | 371 | ORF |
| CBY_1368 | TGTAAA-(28)-TTTACA | 28 | 377 | ORF |
| CBY_3996 | TGTAAA-(7)-TTTACA | 7 | 416 | ORF |
| CBY_1113 | TGTAAA-(19)-TTTACA | 19 | 419 | ORF |
| CBY_3661 | TGTAAA-(16)-TTTACA | 16 | 494 | ORF |
| CBY_2253 | TGTAAA-(19)-TTTACA | 19 | 509 | ORF |
| CBY_3045 | TGTAAA-(39)-TTTACA | 39 | 557 | ORF |
| CBY_0099 | TGTAAA-(16)-TTTACA | 16 | 572 | ORF |
| CBY_1359 | TGTAAA-(7)-TTTACA | 7 | 572 | ORF |
| CBY_1077 | TGTAAA-(28)-TTTACA | 28 | 575 | ORF |
| CBY_0751 | TGTAAA-(16)-TTTACA | 16 | 584 | ORF |
| CBY_2973 | TGTAAA-(6)-TTTACA | 6 | 597 | ORF |
| CBY_0808 | TGTAAA-(36)-TTTACA | 36 | 632 | ORF |
| CBY_3441 | TGTAAA-(0)-TTTACA | 0 | 695 | ORF |
| CBY_2334 | TGTAAA-(25)-TTTACA | 25 | 699 | ORF |
| CBY_0107 | TGTAAA-(7)-TTTACA | 7 | 743 | ORF |
| CBY_2162 | TGTAAA-(38)-TTTACA | 38 | 747 | ORF |
| CBY_0685 | TGTAAA-(36)-TTTACA | 36 | 776 | ORF |
| CBY_1338 | TGTAAA-(19)-TTTACA | 19 | 893 | ORF |
| CBY_3729 | TGTAAA-(7)-TTTACA | 7 | 1094 | ORF |
| CBY_1418 | TGTAAA-(7)-TTTACA | 7 | 1100 | ORF |
| CBY_2968 | TGTAAA-(25)-TTTACA | 25 | 1436 | ORF |
| CBY_0468 | TGTAAA-(9)-TTTACA | 9 | 1521 | ORF |
| CBY_3740 | TGTAAA-(23)-TTTACA | 23 | 1760 | ORF |
| CBY_2237 | TGTAAA-(14)-TTTACA | 14 | 1923 | ORF |
| **4. *Clostridium cellulolyticum* H10** |  |  |  |  |
| Ccel_0922 | TGTAAA-(15)-TTTACA | 15 | -424 | Prom |
| Ccel_1052 | TGTAAA-(36)-TTTACA | 36 | -310 | Prom |
| Ccel_2261 | TGTAAA-(26)-TTTACA | 26 | -308 | Prom |
| Ccel_2764 | TGTAAA-(13)-TTTACA | 13 | -269 | Prom |
| Ccel_2887 | TGTAAA-(27)-TTTACA | 27 | -257 | Prom |
| Ccel_0654 | TGTAAA-(23)-TTTACA | 23 | -253 | Prom |
| Ccel_1051 | TGTAAA-(27)-TTTACA | 27 | -245 | Prom |
| Ccel_2260 | TGTAAA-(26)-TTTACA | 26 | -244 | Prom |
| Ccel_1207 | TGTAAA-(38)-TTTACA | 38 | -218 | Prom |
| Ccel_2790 | TGTAAA-(11)-TTTACA | 11 | -206 | Prom |
| Ccel_2908 | TGTAAA-(25)-TTTACA | 25 | -194 | Prom |
| Ccel_0920 | TGTAAA-(15)-TTTACA | 15 | -182 | Prom |
| Ccel_2681 | TGTAAA-(5)-TTTACA | 5 | -179 | Prom |
| Ccel_2852 | TGTAAA-(34)-TTTACA | 34 | -172 | Prom |
| Ccel_1112 | TGTAAA-(11)-TTTACA | 11 | -133 | Prom |
| Ccel_0092 | TGTAAA-(21)-TTTACA | 21 | -127 | Prom |
| Ccel_2888 | TGTAAA-(27)-TTTACA | 27 | -108 | Prom |
| Ccel_2395 | TGTAAA-(3)-TTTACA | 3 | -107 | Prom |
| Ccel_1974 | TGTAAA-(17)-TTTACA | 17 | -102 | Prom |
| Ccel_0592 | TGTAAA-(3)-TTTACA | 3 | -91 | Prom |
| Ccel_3197 | TGTAAA-(13)-TTTACA | 13 | -80 | Prom |
| Ccel_1751 | TGTAAA-(21)-TTTACA | 21 | -72 | Prom |
| Ccel_2156 | TGTAAA-(12)-TTTACA | 12 | -72 | Prom |
| Ccel_2789 | TGTAAA-(11)-TTTACA | 11 | -66 | Prom |
| Ccel_0655 | TGTAAA-(23)-TTTACA | 23 | -47 | Prom |
| Ccel_2310 | TGTAAA-(33)-TTTACA | 33 | -47 | Prom |
| Ccel_2736 | TGTAAA-(3)-TTTACA | 3 | -31 | Prom |
| Ccel_0728 | TGTAAA-(15)-TTTACA | 15 | -27 | Prom |
| Ccel_0909 | TGTAAA-(25)-TTTACA | 25 | -22 | Prom |
| Ccel_2813 | TGTAAA-(20)-TTTACA | 20 | -8 | Prom |
| Ccel_2444 | TGTAAA-(40)-TTTACA | 40 | -7 | Prom |
| Ccel_1432 | TGTAAA-(15)-TTTACA | 15 | 21 | ORF |
| Ccel_2517 | TGTAAA-(33)-TTTACA | 33 | 44 | ORF |
| Ccel_0414 | TGTAAA-(4)-TTTACA | 4 | 62 | ORF |
| Ccel_1927 | TGTAAA-(33)-TTTACA | 33 | 63 | ORF |
| Ccel_0424 | TGTAAA-(31)-TTTACA | 31 | 77 | ORF |
| Ccel_0564 | TGTAAA-(19)-TTTACA | 19 | 89 | ORF |
| Ccel_0042 | TGTAAA-(8)-TTTACA | 8 | 95 | ORF |
| Ccel_2005 | TGTAAA-(40)-TTTACA | 40 | 125 | ORF |
| Ccel_2143 | TGTAAA-(35)-TTTACA | 35 | 134 | ORF |
| Ccel_2517 | TGTAAA-(25)-TTTACA | 25 | 144 | ORF |
| Ccel_2644 | TGTAAA-(14)-TTTACA | 14 | 146 | ORF |
| Ccel_0864 | TGTAAA-(4)-TTTACA | 4 | 164 | ORF |
| Ccel_0849 | TGTAAA-(24)-TTTACA | 24 | 206 | ORF |
| Ccel_2628 | TGTAAA-(34)-TTTACA | 34 | 212 | ORF |
| Ccel_0277 | TGTAAA-(6)-TTTACA | 6 | 236 | ORF |
| Ccel_1333 | TGTAAA-(9)-TTTACA | 9 | 275 | ORF |
| Ccel_2508 | TGTAAA-(14)-TTTACA | 14 | 311 | ORF |
| Ccel_1064 | TGTAAA-(15)-TTTACA | 15 | 314 | ORF |
| Ccel_2513 | TGTAAA-(27)-TTTACA | 27 | 335 | ORF |
| Ccel_2689 | TGTAAA-(2)-TTTACA | 2 | 344 | ORF |
| Ccel_0072 | TGTAAA-(17)-TTTACA | 17 | 360 | ORF |
| Ccel_3472 | TGTAAA-(24)-TTTACA | 24 | 374 | ORF |
| Ccel_1955 | TGTAAA-(40)-TTTACA | 40 | 389 | ORF |
| Ccel_0317 | TGTAAA-(26)-TTTACA | 26 | 398 | ORF |
| Ccel_2659 | TGTAAA-(34)-TTTACA | 34 | 425 | ORF |
| Ccel_3406 | TGTAAA-(10)-TTTACA | 10 | 443 | ORF |
| Ccel_1628 | TGTAAA-(30)-TTTACA | 30 | 459 | ORF |
| Ccel_2974 | TGTAAA-(11)-TTTACA | 11 | 488 | ORF |
| Ccel_3467 | TGTAAA-(13)-TTTACA | 13 | 491 | ORF |
| Ccel_3019 | TGTAAA-(22)-TTTACA | 22 | 500 | ORF |
| Ccel_1349 | TGTAAA-(40)-TTTACA | 40 | 506 | ORF |
| Ccel_1194 | TGTAAA-(28)-TTTACA | 28 | 532 | ORF |
| Ccel_2270 | TGTAAA-(19)-TTTACA | 19 | 563 | ORF |
| Ccel_1368 | TGTAAA-(38)-TTTACA | 38 | 566 | ORF |
| Ccel_2959 | TGTAAA-(25)-TTTACA | 25 | 572 | ORF |
| Ccel_3325 | TGTAAA-(11)-TTTACA | 11 | 588 | ORF |
| Ccel_0448 | TGTAAA-(28)-TTTACA | 28 | 593 | ORF |
| Ccel_0488 | TGTAAA-(8)-TTTACA | 8 | 626 | ORF |
| Ccel_1709 | TGTAAA-(5)-TTTACA | 5 | 641 | ORF |
| Ccel_1619 | TGTAAA-(16)-TTTACA | 16 | 657 | ORF |
| Ccel_2504 | TGTAAA-(4)-TTTACA | 4 | 680 | ORF |
| Ccel_1749 | TGTAAA-(34)-TTTACA | 34 | 710 | ORF |
| Ccel_0383 | TGTAAA-(40)-TTTACA | 40 | 713 | ORF |
| Ccel_0499 | TGTAAA-(16)-TTTACA | 16 | 716 | ORF |
| Ccel_0613 | TGTAAA-(28)-TTTACA | 28 | 746 | ORF |
| Ccel_1797 | TGTAAA-(13)-TTTACA | 13 | 779 | ORF |
| Ccel_0033 | TGTAAA-(23)-TTTACA | 23 | 782 | ORF |
| Ccel_1370 | TGTAAA-(28)-TTTACA | 28 | 809 | ORF |
| Ccel_1016 | TGTAAA-(22)-TTTACA | 22 | 824 | ORF |
| Ccel_1424 | TGTAAA-(22)-TTTACA | 22 | 860 | ORF |
| Ccel_3142 | TGTAAA-(13)-TTTACA | 13 | 887 | ORF |
| Ccel_0028 | TGTAAA-(25)-TTTACA | 25 | 900 | ORF |
| Ccel_0972 | TGTAAA-(8)-TTTACA | 8 | 962 | ORF |
| Ccel_2333 | TGTAAA-(36)-TTTACA | 36 | 984 | ORF |
| Ccel_2440 | TGTAAA-(14)-TTTACA | 14 | 998 | ORF |
| Ccel_1164 | TGTAAA-(8)-TTTACA | 8 | 1028 | ORF |
| Ccel_1287 | TGTAAA-(24)-TTTACA | 24 | 1031 | ORF |
| Ccel_2606 | TGTAAA-(32)-TTTACA | 32 | 1073 | ORF |
| Ccel_1042 | TGTAAA-(37)-TTTACA | 37 | 1084 | ORF |
| Ccel_3185 | TGTAAA-(36)-TTTACA | 36 | 1160 | ORF |
| Ccel_2452 | TGTAAA-(11)-TTTACA | 11 | 1178 | ORF |
| Ccel_1728 | TGTAAA-(6)-TTTACA | 6 | 1292 | ORF |
| Ccel_3399 | TGTAAA-(14)-TTTACA | 14 | 1343 | ORF |
| Ccel_0251 | TGTAAA-(19)-TTTACA | 19 | 1376 | ORF |
| Ccel_0212 | TGTAAA-(6)-TTTACA | 6 | 1385 | ORF |
| Ccel_2489 | TGTAAA-(34)-TTTACA | 34 | 1406 | ORF |
| Ccel_1234 | TGTAAA-(27)-TTTACA | 27 | 1421 | ORF |
| Ccel_1240 | TGTAAA-(27)-TTTACA | 27 | 1433 | ORF |
| Ccel_1245 | TGTAAA-(36)-TTTACA | 36 | 1535 | ORF |
| Ccel_1855 | TGTAAA-(15)-TTTACA | 15 | 1548 | ORF |
| Ccel_2288 | TGTAAA-(34)-TTTACA | 34 | 1572 | ORF |
| **5. *Clostridium novyi* NT** |  |  |  |  |
| NT01CX_0697 | TGTAAA-(4)-TTTACA | 4 | -485 | Prom |
| NT01CX_0020 | TGTAAA-(18)-TTTACA | 18 | -440 | Prom |
| NT01CX_1238 | TGTAAA-(23)-TTTACA | 23 | -438 | Prom |
| NT01CX_0848 | TGTAAA-(13)-TTTACA | 13 | -331 | Prom |
| NT01CX_0302 | TGTAAA-(8)-TTTACA | 8 | -329 | Prom |
| NT01CX_0140 | TGTAAA-(14)-TTTACA | 14 | -290 | Prom |
| NT01CX_0195 | TGTAAA-(22)-TTTACA | 22 | -269 | Prom |
| NT01CX_1423 | TGTAAA-(15)-TTTACA | 15 | -242 | Prom |
| NT01CX_0734 | TGTAAA-(0)-TTTACA | 0 | -208 | Prom |
| NT01CX_1723 | TGTAAA-(5)-TTTACA | 5 | -196 | Prom |
| NT01CX_0196 | TGTAAA-(22)-TTTACA | 22 | -180 | Prom |
| NT01CX_1829 | TGTAAA-(19)-TTTACA | 19 | -153 | Prom |
| NT01CX_0694 | TGTAAA-(18)-TTTACA | 18 | -143 | Prom |
| NT01CX_0528 | TGTAAA-(13)-TTTACA | 13 | -138 | Prom |
| NT01CX_0150 | TGTAAA-(2)-TTTACA | 2 | -120 | Prom |
| NT01CX_0698 | TGTAAA-(4)-TTTACA | 4 | -118 | Prom |
| NT01CX_0127 | TGTAAA-(11)-TTTACA | 11 | -112 | Prom |
| NT01CX_0733 | TGTAAA-(0)-TTTACA | 0 | -99 | Prom |
| NT01CX_0735 | TGTAAA-(0)-TTTACA | 0 | -99 | Prom |
| NT01CX_0301 | TGTAAA-(8)-TTTACA | 8 | -97 | Prom |
| NT01CX_0693 | TGTAAA-(18)-TTTACA | 18 | -89 | Prom |
| NT01CX_1828 | TGTAAA-(19)-TTTACA | 19 | -77 | Prom |
| NT01CX_0451 | TGTAAA-(6)-TTTACA | 6 | -75 | Prom |
| NT01CX_0126 | TGTAAA-(11)-TTTACA | 11 | -58 | Prom |
| NT01CX_0732 | TGTAAA-(0)-TTTACA | 0 | -47 | Prom |
| NT01CX_1467 | TGTAAA-(18)-TTTACA | 18 | -47 | Prom |
| NT01CX_1500 | TGTAAA-(20)-TTTACA | 20 | -47 | Prom |
| NT01CX_0019 | TGTAAA-(18)-TTTACA | 18 | -46 | Prom |
| NT01CX_0030 | TGTAAA-(7)-TTTACA | 7 | 17 | ORF |
| NT01CX_2346 | TGTAAA-(11)-TTTACA | 11 | 26 | ORF |
| NT01CX_1782 | TGTAAA-(10)-TTTACA | 10 | 38 | ORF |
| NT01CX_0060 | TGTAAA-(21)-TTTACA | 21 | 39 | ORF |
| NT01CX_0001 | TGTAAA-(33)-TTTACA | 33 | 66 | ORF |
| NT01CX_1439 | TGTAAA-(27)-TTTACA | 27 | 77 | ORF |
| NT01CX_0563 | TGTAAA-(31)-TTTACA | 31 | 170 | ORF |
| NT01CX_1602 | TGTAAA-(36)-TTTACA | 36 | 192 | ORF |
| NT01CX_0603 | TGTAAA-(28)-TTTACA | 28 | 209 | ORF |
| NT01CX_0212 | TGTAAA-(40)-TTTACA | 40 | 260 | ORF |
| NT01CX_1282 | TGTAAA-(24)-TTTACA | 24 | 269 | ORF |
| NT01CX_2326 | TGTAAA-(25)-TTTACA | 25 | 269 | ORF |
| NT01CX_0946 | TGTAAA-(37)-TTTACA | 37 | 270 | ORF |
| NT01CX_1962 | TGTAAA-(33)-TTTACA | 33 | 296 | ORF |
| NT01CX_2403 | TGTAAA-(17)-TTTACA | 17 | 296 | ORF |
| NT01CX_1740 | TGTAAA-(27)-TTTACA | 27 | 309 | ORF |
| NT01CX_1889 | TGTAAA-(28)-TTTACA | 28 | 344 | ORF |
| NT01CX_1569 | TGTAAA-(18)-TTTACA | 18 | 348 | ORF |
| NT01CX_1886 | TGTAAA-(10)-TTTACA | 10 | 368 | ORF |
| NT01CX_0308 | TGTAAA-(4)-TTTACA | 4 | 383 | ORF |
| NT01CX_1771 | TGTAAA-(9)-TTTACA | 9 | 426 | ORF |
| NT01CX_0626 | TGTAAA-(37)-TTTACA | 37 | 515 | ORF |
| NT01CX_1776 | TGTAAA-(31)-TTTACA | 31 | 521 | ORF |
| NT01CX_1514 | TGTAAA-(14)-TTTACA | 14 | 536 | ORF |
| NT01CX_2036 | TGTAAA-(25)-TTTACA | 25 | 581 | ORF |
| NT01CX_0394 | TGTAAA-(0)-TTTACA | 0 | 600 | ORF |
| NT01CX_2238 | TGTAAA-(11)-TTTACA | 11 | 726 | ORF |
| NT01CX_1527 | TGTAAA-(28)-TTTACA | 28 | 737 | ORF |
| NT01CX_1319 | TGTAAA-(20)-TTTACA | 20 | 746 | ORF |
| NT01CX_1772 | TGTAAA-(34)-TTTACA | 34 | 971 | ORF |
| NT01CX_0848 | TGTAAA-(12)-TTTACA | 12 | 972 | ORF |
| NT01CX_1320 | TGTAAA-(35)-TTTACA | 35 | 980 | ORF |
| NT01CX_1463 | TGTAAA-(32)-TTTACA | 32 | 995 | ORF |
| NT01CX_1076 | TGTAAA-(29)-TTTACA | 29 | 998 | ORF |
| NT01CX_2104 | TGTAAA-(22)-TTTACA | 22 | 1007 | ORF |
| NT01CX_0205 | TGTAAA-(37)-TTTACA | 37 | 1073 | ORF |
| NT01CX_2254 | TGTAAA-(12)-TTTACA | 12 | 1196 | ORF |
| NT01CX_2417 | TGTAAA-(19)-TTTACA | 19 | 1208 | ORF |
| NT01CX_1594 | TGTAAA-(2)-TTTACA | 2 | 1433 | ORF |
| NT01CX_0598 | TGTAAA-(35)-TTTACA | 35 | 1524 | ORF |
| **6. *Clostridium perfringens* ATCC 13124** |  |  |  |  |
| CPF_0197 | TGTAAA-(3)-TTTACA | 3 | -472 | Prom |
| CPF_1873 | TGTAAA-(38)-TTTACA | 38 | -409 | Prom |
| CPF_0189 | TGTAAA-(38)-TTTACA | 38 | -400 | Prom |
| CPF_1779 | TGTAAA-(40)-TTTACA | 40 | -224 | Prom |
| CPF_0150 | TGTAAA-(13)-TTTACA | 13 | -206 | Prom |
| CPF_0526 | TGTAAA-(16)-TTTACA | 16 | -184 | Prom |
| CPF_2119 | TGTAAA-(19)-TTTACA | 19 | -177 | Prom |
| CPF_1024 | TGTAAA-(20)-TTTACA | 20 | -161 | Prom |
| CPF_0136 | TGTAAA-(29)-TTTACA | 29 | -158 | Prom |
| CPF_1872 | TGTAAA-(38)-TTTACA | 38 | -155 | Prom |
| CPF_1662 | TGTAAA-(26)-TTTACA | 26 | -154 | Prom |
| CPF_0484 | TGTAAA-(20)-TTTACA | 20 | -151 | Prom |
| CPF_1663 | TGTAAA-(26)-TTTACA | 26 | -146 | Prom |
| CPF_2401 | TGTAAA-(31)-TTTACA | 31 | -139 | Prom |
| CPF_2231 | TGTAAA-(15)-TTTACA | 15 | -116 | Prom |
| CPF_1367 | TGTAAA-(37)-TTTACA | 37 | -102 | Prom |
| CPF_1780 | TGTAAA-(40)-TTTACA | 40 | -95 | Prom |
| CPF_2401 | TGTAAA-(20)-TTTACA | 20 | -91 | Prom |
| CPF_2230 | TGTAAA-(15)-TTTACA | 15 | -89 | Prom |
| CPF_0525 | TGTAAA-(16)-TTTACA | 16 | -83 | Prom |
| CPF_2921 | TGTAAA-(31)-TTTACA | 31 | -73 | Prom |
| CPF_1368 | TGTAAA-(37)-TTTACA | 37 | -65 | Prom |
| CPF_2800 | TGTAAA-(32)-TTTACA | 32 | -58 | Prom |
| CPF_1998 | TGTAAA-(28)-TTTACA | 28 | 23 | ORF |
| CPF_1062 | TGTAAA-(17)-TTTACA | 17 | 36 | ORF |
| CPF_1018 | TGTAAA-(36)-TTTACA | 36 | 104 | ORF |
| CPF_1972 | TGTAAA-(36)-TTTACA | 36 | 125 | ORF |
| CPF_0541 | TGTAAA-(7)-TTTACA | 7 | 152 | ORF |
| CPF_0632 | TGTAAA-(24)-TTTACA | 24 | 173 | ORF |
| CPF_1351 | TGTAAA-(4)-TTTACA | 4 | 188 | ORF |
| CPF_0917 | TGTAAA-(23)-TTTACA | 23 | 201 | ORF |
| CPF_0123 | TGTAAA-(1)-TTTACA | 1 | 248 | ORF |
| CPF_2991 | TGTAAA-(26)-TTTACA | 26 | 444 | ORF |
| CPF_1755 | TGTAAA-(28)-TTTACA | 28 | 452 | ORF |
| CPF_2255 | TGTAAA-(10)-TTTACA | 10 | 467 | ORF |
| CPF_0215 | TGTAAA-(39)-TTTACA | 39 | 539 | ORF |
| CPF_0580 | TGTAAA-(9)-TTTACA | 9 | 539 | ORF |
| CPF_2123 | TGTAAA-(9)-TTTACA | 9 | 548 | ORF |
| CPF_1911 | TGTAAA-(5)-TTTACA | 5 | 599 | ORF |
| CPF_1606 | TGTAAA-(2)-TTTACA | 2 | 620 | ORF |
| CPF_1861 | TGTAAA-(21)-TTTACA | 21 | 643 | ORF |
| CPF_0425 | TGTAAA-(34)-TTTACA | 34 | 644 | ORF |
| CPF_1655 | TGTAAA-(9)-TTTACA | 9 | 651 | ORF |
| CPF_1808 | TGTAAA-(22)-TTTACA | 22 | 719 | ORF |
| CPF_0209 | TGTAAA-(10)-TTTACA | 10 | 754 | ORF |
| CPF_2170 | TGTAAA-(37)-TTTACA | 37 | 767 | ORF |
| CPF_2737 | TGTAAA-(34)-TTTACA | 34 | 779 | ORF |
| CPF_1226 | TGTAAA-(6)-TTTACA | 6 | 884 | ORF |
| CPF_1646 | TGTAAA-(7)-TTTACA | 7 | 1025 | ORF |
| CPF_1926 | TGTAAA-(21)-TTTACA | 21 | 1043 | ORF |
| CPF_2320 | TGTAAA-(34)-TTTACA | 34 | 1069 | ORF |
| CPF_0383 | TGTAAA-(18)-TTTACA | 18 | 1302 | ORF |
| **7. *Clostridium tetani* E88** |  |  |  |  |
| CTC01149 | TGTAAA-(24)-TTTACA | 24 | -486 | Prom |
| CTC01291 | TGTAAA-(11)-TTTACA | 11 | -480 | Prom |
| CTC01477 | TGTAAA-(25)-TTTACA | 25 | -417 | Prom |
| CTC02393 | TGTAAA-(32)-TTTACA | 32 | -412 | Prom |
| CTC01299 | TGTAAA-(40)-TTTACA | 40 | -372 | Prom |
| CTC01853 | TGTAAA-(21)-TTTACA | 21 | -367 | Prom |
| CTC00383 | TGTAAA-(12)-TTTACA | 12 | -356 | Prom |
| CTC01116 | TGTAAA-(22)-TTTACA | 22 | -212 | Prom |
| CTC01209 | TGTAAA-(19)-TTTACA | 19 | -203 | Prom |
| CTC02530 | TGTAAA-(39)-TTTACA | 39 | -191 | Prom |
| CTC00228 | TGTAAA-(2)-TTTACA | 2 | -161 | Prom |
| CTC02108 | TGTAAA-(22)-TTTACA | 22 | -143 | Prom |
| CTC01854 | TGTAAA-(21)-TTTACA | 21 | -130 | Prom |
| CTC00878 | TGTAAA-(23)-TTTACA | 23 | -124 | Prom |
| CTC01437 | TGTAAA-(22)-TTTACA | 22 | -91 | Prom |
| CTC00981 | TGTAAA-(7)-TTTACA | 7 | -82 | Prom |
| CTC00982 | TGTAAA-(7)-TTTACA | 7 | -75 | Prom |
| CTC00205 | TGTAAA-(22)-TTTACA | 22 | -70 | Prom |
| CTC01835 | TGTAAA-(26)-TTTACA | 26 | -68 | Prom |
| CTC02503 | TGTAAA-(33)-TTTACA | 33 | -67 | Prom |
| CTC01183 | TGTAAA-(8)-TTTACA | 8 | -64 | Prom |
| CTC01178 | TGTAAA-(25)-TTTACA | 25 | -62 | Prom |
| CTC01168 | TGTAAA-(39)-TTTACA | 39 | 11 | ORF |
| CTC02429 | TGTAAA-(32)-TTTACA | 32 | 27 | ORF |
| CTC00580 | TGTAAA-(1)-TTTACA | 1 | 62 | ORF |
| CTC00170 | TGTAAA-(29)-TTTACA | 29 | 72 | ORF |
| CTC02177 | TGTAAA-(31)-TTTACA | 31 | 119 | ORF |
| CTC00361 | TGTAAA-(15)-TTTACA | 15 | 122 | ORF |
| CTC00696 | TGTAAA-(27)-TTTACA | 27 | 125 | ORF |
| CTC02050 | TGTAAA-(23)-TTTACA | 23 | 170 | ORF |
| CTC02066 | TGTAAA-(28)-TTTACA | 28 | 183 | ORF |
| CTC02554 | TGTAAA-(24)-TTTACA | 24 | 197 | ORF |
| CTC01281 | TGTAAA-(34)-TTTACA | 34 | 203 | ORF |
| CTC00824 | TGTAAA-(27)-TTTACA | 27 | 215 | ORF |
| CTC00272 | TGTAAA-(27)-TTTACA | 27 | 218 | ORF |
| CTC00813 | TGTAAA-(28)-TTTACA | 28 | 335 | ORF |
| CTC01132 | TGTAAA-(37)-TTTACA | 37 | 392 | ORF |
| CTC01742 | TGTAAA-(24)-TTTACA | 24 | 434 | ORF |
| CTC01706 | TGTAAA-(8)-TTTACA | 8 | 459 | ORF |
| CTC00847 | TGTAAA-(18)-TTTACA | 18 | 473 | ORF |
| CTC01745 | TGTAAA-(38)-TTTACA | 38 | 477 | ORF |
| CTC02323 | TGTAAA-(7)-TTTACA | 7 | 509 | ORF |
| CTC01694 | TGTAAA-(29)-TTTACA | 29 | 512 | ORF |
| CTC00684 | TGTAAA-(34)-TTTACA | 34 | 524 | ORF |
| CTC00589 | TGTAAA-(24)-TTTACA | 24 | 537 | ORF |
| CTC00813 | TGTAAA-(8)-TTTACA | 8 | 573 | ORF |
| CTC02012 | TGTAAA-(33)-TTTACA | 33 | 587 | ORF |
| CTC00578 | TGTAAA-(19)-TTTACA | 19 | 620 | ORF |
| CTC01242 | TGTAAA-(19)-TTTACA | 19 | 680 | ORF |
| CTC02195 | TGTAAA-(4)-TTTACA | 4 | 728 | ORF |
| CTC01044 | TGTAAA-(18)-TTTACA | 18 | 758 | ORF |
| CTC01174 | TGTAAA-(28)-TTTACA | 28 | 758 | ORF |
| CTC00489 | TGTAAA-(24)-TTTACA | 24 | 770 | ORF |
| CTC02413 | TGTAAA-(16)-TTTACA | 16 | 782 | ORF |
| CTC02537 | TGTAAA-(9)-TTTACA | 9 | 785 | ORF |
| CTC01086 | TGTAAA-(30)-TTTACA | 30 | 848 | ORF |
| pE88_26 | TGTAAA-(25)-TTTACA | 25 | 851 | ORF |
| CTC02154 | TGTAAA-(7)-TTTACA | 7 | 887 | ORF |
| CTC01279 | TGTAAA-(6)-TTTACA | 6 | 932 | ORF |
| CTC02258 | TGTAAA-(14)-TTTACA | 14 | 956 | ORF |
| CTC02011 | TGTAAA-(24)-TTTACA | 24 | 1136 | ORF |
| pE88_60 | TGTAAA-(26)-TTTACA | 26 | 1145 | ORF |
| CTC00927 | TGTAAA-(10)-TTTACA | 10 | 1236 | ORF |
| CTC01308 | TGTAAA-(12)-TTTACA | 12 | 1376 | ORF |
| CTC01913 | TGTAAA-(25)-TTTACA | 25 | 1484 | ORF |
| CTC00519 | TGTAAA-(37)-TTTACA | 37 | 1538 | ORF |
| **8. *Clostridium difficile* 630** |  |  |  |  |
| CD3205 | TGTAAA-(38)-TTTACA | 38 | -456 | Prom |
| CD3204 | TGTAAA-(38)-TTTACA | 38 | -444 | Prom |
| CD0211 | TGTAAA-(17)-TTTACA | 17 | -401 | Prom |
| CD1340 | TGTAAA-(24)-TTTACA | 24 | -343 | Prom |
| CD3004 | TGTAAA-(35)-TTTACA | 35 | -334 | Prom |
| CD1036 | TGTAAA-(22)-TTTACA | 22 | -321 | Prom |
| CD0623 | TGTAAA-(40)-TTTACA | 40 | -311 | Prom |
| CD0622 | TGTAAA-(40)-TTTACA | 40 | -165 | Prom |
| CD3005 | TGTAAA-(35)-TTTACA | 35 | -294 | Prom |
| CD2499 | TGTAAA-(20)-TTTACA | 20 | -229 | Prom |
| CD0712 | TGTAAA-(25)-TTTACA | 25 | -194 | Prom |
| CD1210 | TGTAAA-(18)-TTTACA | 18 | -190 | Prom |
| CD3230 | TGTAAA-(4)-TTTACA | 4 | -171 | Prom |
| CD3645 | TGTAAA-(27)-TTTACA | 27 | -171 | Prom |
| CD2273 | TGTAAA-(37)-TTTACA | 37 | -162 | Prom |
| CD2272 | TGTAAA-(37)-TTTACA | 37 | -160 | Prom |
| CD0065 | TGTAAA-(2)-TTTACA | 2 | -159 | Prom |
| CD0337 | TGTAAA-(9)-TTTACA | 9 | -153 | Prom |
| CD2783 | TGTAAA-(11)-TTTACA | 11 | -147 | Prom |
| CD1126A | TGTAAA-(14)-TTTACA | 14 | -96 | Prom |
| CD1339 | TGTAAA-(24)-TTTACA | 24 | -77 | Prom |
| CD2549 | TGTAAA-(4)-TTTACA | 4 | -76 | Prom |
| CD0338 | TGTAAA-(9)-TTTACA | 9 | -75 | Prom |
| CD1660 | TGTAAA-(12)-TTTACA | 12 | -73 | Prom |
| CD1803 | TGTAAA-(36)-TTTACA | 36 | -57 | Prom |
| CD0691 | TGTAAA-(7)-TTTACA | 7 | -56 | Prom |
| CD1251A | TGTAAA-(10)-TTTACA | 10 | -46 | Prom |
| CD3227 | TGTAAA-(9)-TTTACA | 9 | 77 | ORF |
| CD3190 | TGTAAA-(25)-TTTACA | 25 | 83 | ORF |
| CD2505 | TGTAAA-(16)-TTTACA | 16 | 96 | ORF |
| CD1408 | TGTAAA-(28)-TTTACA | 28 | 104 | ORF |
| CD3426 | TGTAAA-(35)-TTTACA | 35 | 135 | ORF |
| CD1736 | TGTAAA-(40)-TTTACA | 40 | 137 | ORF |
| CD1462 | TGTAAA-(0)-TTTACA | 0 | 140 | ORF |
| CD1593 | TGTAAA-(28)-TTTACA | 28 | 140 | ORF |
| CD2727 | TGTAAA-(4)-TTTACA | 4 | 146 | ORF |
| CD2622 | TGTAAA-(0)-TTTACA | 0 | 149 | ORF |
| CD3314 | TGTAAA-(35)-TTTACA | 35 | 156 | ORF |
| CD0644 | TGTAAA-(38)-TTTACA | 38 | 170 | ORF |
| CD2150 | TGTAAA-(31)-TTTACA | 31 | 182 | ORF |
| CD0209 | TGTAAA-(19)-TTTACA | 19 | 203 | ORF |
| CD2228 | TGTAAA-(13)-TTTACA | 13 | 215 | ORF |
| CD1367 | TGTAAA-(40)-TTTACA | 40 | 227 | ORF |
| CD0990 | TGTAAA-(0)-TTTACA | 0 | 234 | ORF |
| CD2182 | TGTAAA-(31)-TTTACA | 31 | 237 | ORF |
| CD2590 | TGTAAA-(36)-TTTACA | 36 | 240 | ORF |
| CD2577 | TGTAAA-(28)-TTTACA | 28 | 254 | ORF |
| CD1186 | TGTAAA-(38)-TTTACA | 38 | 296 | ORF |
| CD1026 | TGTAAA-(13)-TTTACA | 13 | 311 | ORF |
| CD0265 | TGTAAA-(40)-TTTACA | 40 | 314 | ORF |
| CD0304 | TGTAAA-(40)-TTTACA | 40 | 314 | ORF |
| CD0972 | TGTAAA-(18)-TTTACA | 18 | 315 | ORF |
| CD2894 | TGTAAA-(18)-TTTACA | 18 | 315 | ORF |
| CD1792 | TGTAAA-(3)-TTTACA | 3 | 327 | ORF |
| CD3316 | TGTAAA-(29)-TTTACA | 29 | 338 | ORF |
| CD2950 | TGTAAA-(39)-TTTACA | 39 | 347 | ORF |
| CD3211 | TGTAAA-(9)-TTTACA | 9 | 354 | ORF |
| CD1463 | TGTAAA-(12)-TTTACA | 12 | 359 | ORF |
| CD1415 | TGTAAA-(25)-TTTACA | 25 | 362 | ORF |
| CD1373 | TGTAAA-(6)-TTTACA | 6 | 368 | ORF |
| CD3669 | TGTAAA-(28)-TTTACA | 28 | 377 | ORF |
| CD0166 | TGTAAA-(2)-TTTACA | 2 | 387 | ORF |
| CD1374 | TGTAAA-(22)-TTTACA | 22 | 392 | ORF |
| CD1968 | TGTAAA-(31)-TTTACA | 31 | 395 | ORF |
| CD3552 | TGTAAA-(2)-TTTACA | 2 | 426 | ORF |
| CD0418 | TGTAAA-(37)-TTTACA | 37 | 434 | ORF |
| CD1297 | TGTAAA-(7)-TTTACA | 7 | 440 | ORF |
| CD0430 | TGTAAA-(18)-TTTACA | 18 | 467 | ORF |
| CD2817 | TGTAAA-(37)-TTTACA | 37 | 467 | ORF |
| CD3096 | TGTAAA-(16)-TTTACA | 16 | 467 | ORF |
| CD1856 | TGTAAA-(37)-TTTACA | 37 | 470 | ORF |
| CD2435 | TGTAAA-(2)-TTTACA | 2 | 474 | ORF |
| CD2278 | TGTAAA-(13)-TTTACA | 13 | 491 | ORF |
| CD2876 | TGTAAA-(40)-TTTACA | 40 | 494 | ORF |
| CD2382 | TGTAAA-(25)-TTTACA | 25 | 506 | ORF |
| CD1654 | TGTAAA-(27)-TTTACA | 27 | 518 | ORF |
| CD1432 | TGTAAA-(1)-TTTACA | 1 | 527 | ORF |
| CD1583 | TGTAAA-(18)-TTTACA | 18 | 552 | ORF |
| CD2842 | TGTAAA-(2)-TTTACA | 2 | 566 | ORF |
| CD0057 | TGTAAA-(15)-TTTACA | 15 | 587 | ORF |
| CD0696 | TGTAAA-(4)-TTTACA | 4 | 590 | ORF |
| CD1859 | TGTAAA-(40)-TTTACA | 40 | 590 | ORF |
| CD3069 | TGTAAA-(34)-TTTACA | 34 | 599 | ORF |
| CD1028 | TGTAAA-(21)-TTTACA | 21 | 743 | ORF |
| CD2811 | TGTAAA-(4)-TTTACA | 4 | 749 | ORF |
| CD2248 | TGTAAA-(37)-TTTACA | 37 | 794 | ORF |
| CD3414 | TGTAAA-(19)-TTTACA | 19 | 803 | ORF |
| CD1084 | TGTAAA-(34)-TTTACA | 34 | 836 | ORF |
| CD0113 | TGTAAA-(40)-TTTACA | 40 | 893 | ORF |
| CD1696 | TGTAAA-(28)-TTTACA | 28 | 896 | ORF |
| CD0014 | TGTAAA-(22)-TTTACA | 22 | 956 | ORF |
| CD2963 | TGTAAA-(29)-TTTACA | 29 | 965 | ORF |
| CD1018 | TGTAAA-(37)-TTTACA | 37 | 1004 | ORF |
| CD2669a | TGTAAA-(39)-TTTACA | 39 | 1101 | ORF |
| CD3599 | TGTAAA-(0)-TTTACA | 0 | 1103 | ORF |
| CD3659 | TGTAAA-(26)-TTTACA | 26 | 1122 | ORF |
| CD2981 | TGTAAA-(39)-TTTACA | 39 | 1248 | ORF |
| CD2503 | TGTAAA-(25)-TTTACA | 25 | 1301 | ORF |
| CD2956 | TGTAAA-(39)-TTTACA | 39 | 1343 | ORF |
| CD0283 | TGTAAA-(6)-TTTACA | 6 | 1484 | ORF |
| CD0033 | TGTAAA-(9)-TTTACA | 9 | 1521 | ORF |
| CD1591 | TGTAAA-(31)-TTTACA | 31 | 1547 | ORF |
| CD0039 | TGTAAA-(9)-TTTACA | 9 | 1694 | ORF |
| CD3411 | TGTAAA-(16)-TTTACA | 16 | 1883 | ORF |
| **9. *Clostridium thermocellum* ATCC 27405** |  |  |  |  |
| Cthe_1988 | TGTAAA-(2)-TTTACA | 2 | -428 | Prom |
| Cthe_3027 | TGTAAA-(22)-TTTACA | 22 | -390 | Prom |
| Cthe_1266 | TGTAAA-(35)-TTTACA | 35 | -330 | Prom |
| Cthe_3035 | TGTAAA-(13)-TTTACA | 13 | -328 | Prom |
| Cthe_3036 | TGTAAA-(13)-TTTACA | 13 | -313 | Prom |
| Cthe_2285 | TGTAAA-(32)-TTTACA | 32 | -311 | Prom |
| Cthe_1695 | TGTAAA-(19)-TTTACA | 19 | -284 | Prom |
| Cthe_2004 | TGTAAA-(2)-TTTACA | 2 | -277 | Prom |
| Cthe_2973 | TGTAAA-(39)-TTTACA | 39 | -271 | Prom |
| Cthe_0083 | TGTAAA-(18)-TTTACA | 18 | -203 | Prom |
| Cthe_2625 | TGTAAA-(26)-TTTACA | 26 | -155 | Prom |
| Cthe_2972 | TGTAAA-(39)-TTTACA | 39 | -151 | Prom |
| Cthe_3026 | TGTAAA-(22)-TTTACA | 22 | -147 | Prom |
| Cthe_1880 | TGTAAA-(7)-TTTACA | 7 | -129 | Prom |
| Cthe_0967 | TGTAAA-(22)-TTTACA | 22 | -117 | Prom |
| Cthe_0153 | TGTAAA-(2)-TTTACA | 2 | -88 | Prom |
| Cthe_2177 | TGTAAA-(9)-TTTACA | 9 | -84 | Prom |
| Cthe_0131 | TGTAAA-(19)-TTTACA | 19 | -77 | Prom |
| Cthe_0976 | TGTAAA-(29)-TTTACA | 29 | -72 | Prom |
| Cthe_1120 | TGTAAA-(7)-TTTACA | 7 | -72 | Prom |
| Cthe_1879 | TGTAAA-(7)-TTTACA | 7 | -72 | Prom |
| Cthe_3231 | TGTAAA-(24)-TTTACA | 24 | -66 | Prom |
| Cthe_3371 | TGTAAA-(19)-TTTACA | 19 | -64 | Prom |
| Cthe_0771 | TGTAAA-(10)-TTTACA | 10 | -31 | Prom |
| Cthe_0183 | TGTAAA-(38)-TTTACA | 38 | 47 | ORF |
| Cthe_0616 | TGTAAA-(40)-TTTACA | 40 | 143 | ORF |
| Cthe_2571 | TGTAAA-(40)-TTTACA | 40 | 144 | ORF |
| Cthe_2397 | TGTAAA-(8)-TTTACA | 8 | 146 | ORF |
| Cthe_2211 | TGTAAA-(25)-TTTACA | 25 | 167 | ORF |
| Cthe_2248 | TGTAAA-(23)-TTTACA | 23 | 197 | ORF |
| Cthe_0258 | TGTAAA-(28)-TTTACA | 28 | 221 | ORF |
| Cthe_0519 | TGTAAA-(34)-TTTACA | 34 | 234 | ORF |
| Cthe_0349 | TGTAAA-(18)-TTTACA | 18 | 267 | ORF |
| Cthe_1998 | TGTAAA-(2)-TTTACA | 2 | 353 | ORF |
| Cthe_0646 | TGTAAA-(28)-TTTACA | 28 | 383 | ORF |
| Cthe_2079 | TGTAAA-(11)-TTTACA | 11 | 407 | ORF |
| Cthe_0717 | TGTAAA-(14)-TTTACA | 14 | 413 | ORF |
| Cthe_1294 | TGTAAA-(38)-TTTACA | 38 | 413 | ORF |
| Cthe_2006 | TGTAAA-(31)-TTTACA | 31 | 428 | ORF |
| Cthe_1063 | TGTAAA-(37)-TTTACA | 37 | 434 | ORF |
| Cthe_3043 | TGTAAA-(14)-TTTACA | 14 | 455 | ORF |
| Cthe_2807 | TGTAAA-(32)-TTTACA | 32 | 467 | ORF |
| Cthe_2053 | TGTAAA-(10)-TTTACA | 10 | 497 | ORF |
| Cthe_2549 | TGTAAA-(37)-TTTACA | 37 | 497 | ORF |
| Cthe_2863 | TGTAAA-(2)-TTTACA | 2 | 518 | ORF |
| Cthe_1167 | TGTAAA-(17)-TTTACA | 17 | 557 | ORF |
| Cthe_1165 | TGTAAA-(14)-TTTACA | 14 | 569 | ORF |
| Cthe_0561 | TGTAAA-(31)-TTTACA | 31 | 575 | ORF |
| Cthe_1696 | TGTAAA-(38)-TTTACA | 38 | 579 | ORF |
| Cthe_1534 | TGTAAA-(35)-TTTACA | 35 | 590 | ORF |
| Cthe_1286 | TGTAAA-(31)-TTTACA | 31 | 611 | ORF |
| Cthe_1417 | TGTAAA-(36)-TTTACA | 36 | 650 | ORF |
| Cthe_1151 | TGTAAA-(14)-TTTACA | 14 | 656 | ORF |
| Cthe_2890 | TGTAAA-(5)-TTTACA | 5 | 674 | ORF |
| Cthe_3114 | TGTAAA-(17)-TTTACA | 17 | 701 | ORF |
| Cthe_0848 | TGTAAA-(2)-TTTACA | 2 | 728 | ORF |
| Cthe_1958 | TGTAAA-(15)-TTTACA | 15 | 797 | ORF |
| Cthe_1926 | TGTAAA-(2)-TTTACA | 2 | 833 | ORF |
| Cthe_1379 | TGTAAA-(20)-TTTACA | 20 | 851 | ORF |
| Cthe_2612 | TGTAAA-(14)-TTTACA | 14 | 884 | ORF |
| Cthe_2737 | TGTAAA-(5)-TTTACA | 5 | 899 | ORF |
| Cthe_1041 | TGTAAA-(2)-TTTACA | 2 | 989 | ORF |
| Cthe_0760 | TGTAAA-(5)-TTTACA | 5 | 1088 | ORF |
| Cthe_1706 | TGTAAA-(19)-TTTACA | 19 | 1122 | ORF |
| Cthe_1028 | TGTAAA-(25)-TTTACA | 25 | 1184 | ORF |
| Cthe_2030 | TGTAAA-(2)-TTTACA | 2 | 1259 | ORF |
| Cthe_2834 | TGTAAA-(2)-TTTACA | 2 | 1259 | ORF |
| Cthe_1989 | TGTAAA-(2)-TTTACA | 2 | 1260 | ORF |
| Cthe_2570 | TGTAAA-(25)-TTTACA | 25 | 1749 | ORF |
| Cthe_2346 | TGTAAA-(35)-TTTACA | 35 | 1919 | ORF |
| **The *cre_var_* sites in class Bacilli** |  |  |  |  |
| 1. ***Bacillus*** |  |  |  |  |
| 1. ***Bacillus subtilis* subsp. *Subtilis* str. 168** |  |  |  |  |
| BSU33190 | TGTAAA-(20)-TTTACA | 20 | -344 | Prom |
| BSU35070 | TGTAAA-(21)-TTTACA | 21 | -276 | Prom |
| BSU04530 | TGTAAA-(27)-TTTACA | 27 | -233 | Prom |
| BSU14580 | TGTAAA-(32)-TTTACA | 32 | -226 | Prom |
| BSU11270 | TGTAAA-(34)-TTTACA | 34 | -206 | Prom |
| BSU33840 | TGTAAA-(8)-TTTACA | 8 | -196 | Prom |
| BSU35080 | TGTAAA-(21)-TTTACA | 21 | -182 | Prom |
| BSU22720 | TGTAAA-(2)-TTTACA | 2 | -175 | Prom |
| BSU04540 | TGTAAA-(27)-TTTACA | 27 | -170 | Prom |
| BSU19180 | TGTAAA-(4)-TTTACA | 4 | -155 | Prom |
| BSU00700 | TGTAAA-(20)-TTTACA | 20 | -145 | Prom |
| BSU11280 | TGTAAA-(34)-TTTACA | 34 | -88 | Prom |
| BSU33180 | TGTAAA-(20)-TTTACA | 20 | -85 | Prom |
| BSU25190 | TGTAAA-(21)-TTTACA | 21 | -64 | Prom |
| BSU19630 | TGTAAA-(26)-TTTACA | 26 | -50 | Prom |
| BSU39320 | TGTAAA-(3)-TTTACA | 3 | -45 | Prom |
| BSU07640 | TGTAAA-(32)-TTTACA | 32 | -43 | Prom |
| BSU32150 | TGTAAA-(24)-TTTACA | 24 | 5 | ORF |
| BSU20080 | TGTAAA-(19)-TTTACA | 19 | 38 | ORF |
| BSU39880 | TGTAAA-(31)-TTTACA | 31 | 80 | ORF |
| BSU27620 | TGTAAA-(36)-TTTACA | 36 | 104 | ORF |
| BSU36050 | TGTAAA-(15)-TTTACA | 15 | 131 | ORF |
| BSU05910 | TGTAAA-(10)-TTTACA | 10 | 170 | ORF |
| BSU00610 | TGTAAA-(17)-TTTACA | 17 | 185 | ORF |
| BSU02200 | TGTAAA-(18)-TTTACA | 18 | 267 | ORF |
| BSU32020 | TGTAAA-(21)-TTTACA | 21 | 296 | ORF |
| BSU17650 | TGTAAA-(35)-TTTACA | 35 | 348 | ORF |
| BSU00910 | TGTAAA-(25)-TTTACA | 25 | 386 | ORF |
| BSU10020 | TGTAAA-(12)-TTTACA | 12 | 410 | ORF |
| BSU10790 | TGTAAA-(8)-TTTACA | 8 | 434 | ORF |
| BSU21140 | TGTAAA-(17)-TTTACA | 17 | 464 | ORF |
| BSU37500 | TGTAAA-(4)-TTTACA | 4 | 491 | ORF |
| BSU16750 | TGTAAA-(5)-TTTACA | 5 | 497 | ORF |
| BSU17760 | TGTAAA-(16)-TTTACA | 16 | 563 | ORF |
| BSU11250 | TGTAAA-(28)-TTTACA | 28 | 659 | ORF |
| BSU37660 | TGTAAA-(32)-TTTACA | 32 | 893 | ORF |
| BSU18340 | TGTAAA-(24)-TTTACA | 24 | 989 | ORF |
| BSU13010 | TGTAAA-(3)-TTTACA | 3 | 1025 | ORF |
| BSU08790 | TGTAAA-(10)-TTTACA | 10 | 1233 | ORF |
| 1. ***Bacillus cereus* ATCC 14579** |  |  |  |  |
| BC3487 | TGTAAA-(27)-TTTACA | 27 | -459 | Prom |
| BC2867 | TGTAAA-(7)-TTTACA | 7 | -431 | Prom |
| BC4142 | TGTAAA-(10)-TTTACA | 10 | -431 | Prom |
| BC2735 | TGTAAA-(14)-TTTACA | 14 | -398 | Prom |
| BC4978 | TGTAAA-(23)-TTTACA | 23 | -362 | Prom |
| BC3439 | TGTAAA-(19)-TTTACA | 19 | -354 | Prom |
| BC1936 | TGTAAA-(30)-TTTACA | 30 | -343 | Prom |
| BC3439 | TGTAAA-(8)-TTTACA | 8 | -343 | Prom |
| BC1709 | TGTAAA-(38)-TTTACA | 38 | -326 | Prom |
| BC2118 | TGTAAA-(40)-TTTACA | 40 | -320 | Prom |
| BC2338 | TGTAAA-(19)-TTTACA | 19 | -311 | Prom |
| BC0709 | TGTAAA-(37)-TTTACA | 37 | -281 | Prom |
| BC2956 | TGTAAA-(6)-TTTACA | 6 | -254 | Prom |
| BC4349 | TGTAAA-(6)-TTTACA | 6 | -252 | Prom |
| BC0710 | TGTAAA-(37)-TTTACA | 37 | -250 | Prom |
| BC1955 | TGTAAA-(15)-TTTACA | 15 | -244 | Prom |
| BC1031 | TGTAAA-(35)-TTTACA | 35 | -242 | Prom |
| BC2117 | TGTAAA-(40)-TTTACA | 40 | -236 | Prom |
| BC4977 | TGTAAA-(23)-TTTACA | 23 | -228 | Prom |
| BC1697 | TGTAAA-(4)-TTTACA | 4 | -224 | Prom |
| BC3874 | TGTAAA-(18)-TTTACA | 18 | -204 | Prom |
| BC2745 | TGTAAA-(6)-TTTACA | 6 | -188 | Prom |
| BC1954 | TGTAAA-(26)-TTTACA | 26 | -179 | Prom |
| BC5431 | TGTAAA-(9)-TTTACA | 9 | -166 | Prom |
| BC3586 | TGTAAA-(34)-TTTACA | 34 | -165 | Prom |
| BC2603 | TGTAAA-(30)-TTTACA | 30 | -152 | Prom |
| BC3857 | TGTAAA-(30)-TTTACA | 30 | -136 | Prom |
| BC3875 | TGTAAA-(18)-TTTACA | 18 | -132 | Prom |
| BC1252 | TGTAAA-(31)-TTTACA | 31 | -129 | Prom |
| BC4814 | TGTAAA-(36)-TTTACA | 36 | -121 | Prom |
| BC5250 | TGTAAA-(13)-TTTACA | 13 | -113 | Prom |
| BC0354 | TGTAAA-(29)-TTTACA | 29 | -109 | Prom |
| BC4348 | TGTAAA-(6)-TTTACA | 6 | -101 | Prom |
| BC3532 | TGTAAA-(15)-TTTACA | 15 | -96 | Prom |
| BC0059 | TGTAAA-(25)-TTTACA | 25 | -91 | Prom |
| BC4815 | TGTAAA-(36)-TTTACA | 36 | -88 | Prom |
| BC0803 | TGTAAA-(16)-TTTACA | 16 | -85 | Prom |
| BC5178 | TGTAAA-(24)-TTTACA | 24 | -84 | Prom |
| BC2551 | TGTAAA-(14)-TTTACA | 14 | -81 | Prom |
| BC4784 | TGTAAA-(6)-TTTACA | 6 | -77 | Prom |
| BC1253 | TGTAAA-(22)-TTTACA | 22 | -70 | Prom |
| BC1954 | TGTAAA-(14)-TTTACA | 14 | -66 | Prom |
| BC1364 | TGTAAA-(15)-TTTACA | 15 | -54 | Prom |
| BC4184 | TGTAAA-(30)-TTTACA | 30 | -41 | Prom |
| BC1698 | TGTAAA-(4)-TTTACA | 4 | -38 | Prom |
| BC2746 | TGTAAA-(6)-TTTACA | 6 | -38 | Prom |
| BC0435 | TGTAAA-(6)-TTTACA | 6 | -34 | Prom |
| BC4784 | TGTAAA-(6)-TTTACA | 6 | -34 | Prom |
| BC2140 | TGTAAA-(29)-TTTACA | 29 | -24 | Prom |
| BC3011 | TGTAAA-(14)-TTTACA | 14 | -7 | Prom |
| BC3765 | TGTAAA-(18)-TTTACA | 18 | -7 | Prom |
| BC2275 | TGTAAA-(31)-TTTACA | 31 | 8 | ORF |
| BC3655 | TGTAAA-(24)-TTTACA | 24 | 8 | ORF |
| BC2774 | TGTAAA-(33)-TTTACA | 33 | 42 | ORF |
| BC1575 | TGTAAA-(6)-TTTACA | 6 | 50 | ORF |
| BC2891 | TGTAAA-(27)-TTTACA | 27 | 53 | ORF |
| BC1196 | TGTAAA-(18)-TTTACA | 18 | 63 | ORF |
| BC0003 | TGTAAA-(6)-TTTACA | 6 | 83 | ORF |
| BC3092 | TGTAAA-(30)-TTTACA | 30 | 86 | ORF |
| BC4671 | TGTAAA-(0)-TTTACA | 0 | 92 | ORF |
| BC1339 | TGTAAA-(33)-TTTACA | 33 | 95 | ORF |
| BC2200 | TGTAAA-(27)-TTTACA | 27 | 95 | ORF |
| BC3972 | TGTAAA-(27)-TTTACA | 27 | 95 | ORF |
| BC3945 | TGTAAA-(19)-TTTACA | 19 | 104 | ORF |
| BC5260 | TGTAAA-(15)-TTTACA | 15 | 104 | ORF |
| BC2783 | TGTAAA-(3)-TTTACA | 3 | 122 | ORF |
| BC1344 | TGTAAA-(16)-TTTACA | 16 | 131 | ORF |
| BC3060 | TGTAAA-(24)-TTTACA | 24 | 146 | ORF |
| BC1033 | TGTAAA-(8)-TTTACA | 8 | 147 | ORF |
| BC2774 | TGTAAA-(30)-TTTACA | 30 | 159 | ORF |
| BC2431 | TGTAAA-(7)-TTTACA | 7 | 206 | ORF |
| BC0410 | TGTAAA-(31)-TTTACA | 31 | 209 | ORF |
| BC4046 | TGTAAA-(29)-TTTACA | 29 | 214 | ORF |
| BC4009 | TGTAAA-(12)-TTTACA | 12 | 230 | ORF |
| BC0037 | TGTAAA-(39)-TTTACA | 39 | 248 | ORF |
| BC5487 | TGTAAA-(16)-TTTACA | 16 | 251 | ORF |
| BC2198 | TGTAAA-(6)-TTTACA | 6 | 260 | ORF |
| BC0650 | TGTAAA-(16)-TTTACA | 16 | 263 | ORF |
| BC1223 | TGTAAA-(34)-TTTACA | 34 | 266 | ORF |
| BC4515 | TGTAAA-(30)-TTTACA | 30 | 278 | ORF |
| BC3222 | TGTAAA-(11)-TTTACA | 11 | 305 | ORF |
| BC4092 | TGTAAA-(16)-TTTACA | 16 | 314 | ORF |
| BC2020 | TGTAAA-(14)-TTTACA | 14 | 315 | ORF |
| BC3515 | TGTAAA-(3)-TTTACA | 3 | 317 | ORF |
| BC3774 | TGTAAA-(16)-TTTACA | 16 | 320 | ORF |
| BC5204 | TGTAAA-(5)-TTTACA | 5 | 320 | ORF |
| BC5373 | TGTAAA-(18)-TTTACA | 18 | 338 | ORF |
| BC2320 | TGTAAA-(21)-TTTACA | 21 | 341 | ORF |
| BC5000 | TGTAAA-(15)-TTTACA | 15 | 356 | ORF |
| BC0107 | TGTAAA-(25)-TTTACA | 25 | 386 | ORF |
| BC3062 | TGTAAA-(11)-TTTACA | 11 | 399 | ORF |
| BCp0013 | TGTAAA-(22)-TTTACA | 22 | 404 | ORF |
| BC0103 | TGTAAA-(33)-TTTACA | 33 | 425 | ORF |
| BC3099 | TGTAAA-(36)-TTTACA | 36 | 434 | ORF |
| BC3656 | TGTAAA-(20)-TTTACA | 20 | 438 | ORF |
| BC1697 | TGTAAA-(8)-TTTACA | 8 | 440 | ORF |
| BC3476 | TGTAAA-(11)-TTTACA | 11 | 447 | ORF |
| BC4693 | TGTAAA-(31)-TTTACA | 31 | 455 | ORF |
| BC3548 | TGTAAA-(4)-TTTACA | 4 | 479 | ORF |
| BC0510 | TGTAAA-(34)-TTTACA | 34 | 485 | ORF |
| BC0738 | TGTAAA-(30)-TTTACA | 30 | 486 | ORF |
| BC0756 | TGTAAA-(27)-TTTACA | 27 | 491 | ORF |
| BC1365 | TGTAAA-(0)-TTTACA | 0 | 494 | ORF |
| BC4033 | TGTAAA-(18)-TTTACA | 18 | 495 | ORF |
| BC5252 | TGTAAA-(25)-TTTACA | 25 | 497 | ORF |
| BC3245 | TGTAAA-(10)-TTTACA | 10 | 512 | ORF |
| BC4449 | TGTAAA-(22)-TTTACA | 22 | 521 | ORF |
| BC0324 | TGTAAA-(6)-TTTACA | 6 | 638 | ORF |
| BC1325 | TGTAAA-(31)-TTTACA | 31 | 638 | ORF |
| BC0493 | TGTAAA-(33)-TTTACA | 33 | 650 | ORF |
| BC2652 | TGTAAA-(17)-TTTACA | 17 | 662 | ORF |
| BC2832 | TGTAAA-(25)-TTTACA | 25 | 692 | ORF |
| BC4702 | TGTAAA-(27)-TTTACA | 27 | 695 | ORF |
| BC4647 | TGTAAA-(24)-TTTACA | 24 | 702 | ORF |
| BC2676 | TGTAAA-(34)-TTTACA | 34 | 719 | ORF |
| BC0659 | TGTAAA-(36)-TTTACA | 36 | 734 | ORF |
| BC0629 | TGTAAA-(38)-TTTACA | 38 | 737 | ORF |
| BC2326 | TGTAAA-(22)-TTTACA | 22 | 737 | ORF |
| BC4208 | TGTAAA-(12)-TTTACA | 12 | 776 | ORF |
| BC1112 | TGTAAA-(27)-TTTACA | 27 | 785 | ORF |
| BC4372 | TGTAAA-(36)-TTTACA | 36 | 806 | ORF |
| BC2296 | TGTAAA-(15)-TTTACA | 15 | 845 | ORF |
| BC4303 | TGTAAA-(22)-TTTACA | 22 | 899 | ORF |
| BC0426 | TGTAAA-(7)-TTTACA | 7 | 953 | ORF |
| BC0351 | TGTAAA-(14)-TTTACA | 14 | 1001 | ORF |
| BC1783 | TGTAAA-(30)-TTTACA | 30 | 1001 | ORF |
| BC2998 | TGTAAA-(39)-TTTACA | 39 | 1019 | ORF |
| BC1138 | TGTAAA-(4)-TTTACA | 4 | 1055 | ORF |
| BC1193 | TGTAAA-(7)-TTTACA | 7 | 1157 | ORF |
| BC1725 | TGTAAA-(11)-TTTACA | 11 | 1185 | ORF |
| BC3787 | TGTAAA-(36)-TTTACA | 36 | 1208 | ORF |
| BC0258 | TGTAAA-(33)-TTTACA | 33 | 1235 | ORF |
| BC5412 | TGTAAA-(16)-TTTACA | 16 | 1358 | ORF |
| BC4050 | TGTAAA-(34)-TTTACA | 34 | 1934 | ORF |
| BC1908 | TGTAAA-(12)-TTTACA | 12 | 1955 | ORF |
| **2. *Streptococcus*** |  |  |  |  |
| 1. ***Streptococcus gordonii* str. *Challis* substr** |  |  |  |  |
| SGO_0723 | TGTAAA-(31)-TTTACA | 31 | -141 | Prom |
| SGO_0549 | TGTAAA-(14)-TTTACA | 14 | -122 | Prom |
| SGO_1637 | TGTAAA-(14)-TTTACA | 14 | -89 | Prom |
| SGO_1077 | TGTAAA-(3)-TTTACA | 3 | -74 | Prom |
| SGO_1906 | TGTAAA-(34)-TTTACA | 34 | -65 | Prom |
| SGO_0289 | TGTAAA-(27)-TTTACA | 27 | -64 | Prom |
| SGO_1078 | TGTAAA-(3)-TTTACA | 3 | -45 | Prom |
| SGO_1638 | TGTAAA-(14)-TTTACA | 14 | -34 | Prom |
| SGO_1754 | TGTAAA-(12)-TTTACA | 12 | 14 | ORF |
| SGO_1754 | TGTAAA-(24)-TTTACA | 24 | 56 | ORF |
| SGO_0109 | TGTAAA-(18)-TTTACA | 18 | 62 | ORF |
| SGO_2141 | TGTAAA-(15)-TTTACA | 15 | 90 | ORF |
| SGO_1838 | TGTAAA-(6)-TTTACA | 6 | 125 | ORF |
| SGO_1100 | TGTAAA-(35)-TTTACA | 35 | 323 | ORF |
| SGO_1860 | TGTAAA-(13)-TTTACA | 13 | 341 | ORF |
| SGO_1959 | TGTAAA-(16)-TTTACA | 16 | 353 | ORF |
| SGO_2146 | TGTAAA-(17)-TTTACA | 17 | 377 | ORF |
| SGO_2141 | TGTAAA-(6)-TTTACA | 6 | 509 | ORF |
| SGO_1218 | TGTAAA-(18)-TTTACA | 18 | 647 | ORF |
| SGO_1307 | TGTAAA-(19)-TTTACA | 19 | 713 | ORF |
| SGO_0641 | TGTAAA-(22)-TTTACA | 22 | 725 | ORF |
| SGO_0144 | TGTAAA-(12)-TTTACA | 12 | 731 | ORF |
| SGO_1712 | TGTAAA-(23)-TTTACA | 23 | 1214 | ORF |
| SGO_0556 | TGTAAA-(13)-TTTACA | 13 | 1763 | ORF |
| 1. ***Streptococcus pneumoniae* TIGR4** |  |  |  |  |
| SP_0828 | TGTAAA-(25)-TTTACA | 25 | -186 | Prom |
| SP_1187 | TGTAAA-(2)-TTTACA | 2 | -179 | Prom |
| SP_1529 | TGTAAA-(14)-TTTACA | 14 | -129 | Prom |
| SP_2229 | TGTAAA-(5)-TTTACA | 5 | -127 | Prom |
| SP_2230 | TGTAAA-(5)-TTTACA | 5 | -95 | Prom |
| SP_0108 | TGTAAA-(1)-TTTACA | 1 | -76 | Prom |
| SP_0114 | TGTAAA-(1)-TTTACA | 1 | -76 | Prom |
| SP_0142 | TGTAAA-(33)-TTTACA | 33 | -73 | Prom |
| SP_2240 | TGTAAA-(6)-TTTACA | 6 | -47 | Prom |
| SP_0841 | TGTAAA-(3)-TTTACA | 3 | -45 | Prom |
| SP_1530 | TGTAAA-(14)-TTTACA | 14 | -35 | Prom |
| SP_1109 | TGTAAA-(1)-TTTACA | 1 | -34 | Prom |
| SP_0122 | TGTAAA-(18)-TTTACA | 18 | -20 | Prom |
| SP_0142 | TGTAAA-(10)-TTTACA | 10 | -8 | Prom |
| SP_0820 | TGTAAA-(22)-TTTACA | 22 | 15 | ORF |
| SP_1242 | TGTAAA-(3)-TTTACA | 3 | 20 | ORF |
| SP_0646 | TGTAAA-(28)-TTTACA | 28 | 125 | ORF |
| SP_1197 | TGTAAA-(28)-TTTACA | 28 | 125 | ORF |
| SP_1836 | TGTAAA-(25)-TTTACA | 25 | 131 | ORF |
| SP_1057 | TGTAAA-(29)-TTTACA | 29 | 302 | ORF |
| SP_0236 | TGTAAA-(16)-TTTACA | 16 | 353 | ORF |
| SP_1897 | TGTAAA-(8)-TTTACA | 8 | 386 | ORF |
| SP_0745 | TGTAAA-(34)-TTTACA | 34 | 497 | ORF |
| SP_0005 | TGTAAA-(6)-TTTACA | 6 | 509 | ORF |
| SP_1467 | TGTAAA-(31)-TTTACA | 31 | 564 | ORF |
| SP_1319 | TGTAAA-(36)-TTTACA | 36 | 599 | ORF |
| SP_1062 | TGTAAA-(11)-TTTACA | 11 | 600 | ORF |
| SP_0585 | TGTAAA-(38)-TTTACA | 38 | 626 | ORF |
| SP_1890 | TGTAAA-(40)-TTTACA | 40 | 1355 | ORF |
| 1. ***Streptococcus pyogenes* M1 GAS** |  |  |  |  |
| SPy1912 | TGTAAA-(33)-TTTACA | 33 | -225 | Prom |
| SPy2169 | TGTAAA-(5)-TTTACA | 5 | -190 | Prom |
| SPy2159 | TGTAAA-(28)-TTTACA | 28 | -171 | Prom |
| SPy2157 | TGTAAA-(28)-TTTACA | 28 | -150 | Prom |
| SPy0379 | TGTAAA-(4)-TTTACA | 4 | -34 | Prom |
| SPy1288 | TGTAAA-(11)-TTTACA | 11 | 107 | ORF |
| SPy0484 | TGTAAA-(39)-TTTACA | 39 | 114 | ORF |
| SPy0369 | TGTAAA-(35)-TTTACA | 35 | 119 | ORF |
| SPy0337 | TGTAAA-(37)-TTTACA | 37 | 170 | ORF |
| SPy1622 | TGTAAA-(27)-TTTACA | 27 | 383 | ORF |
| SPy1477 | TGTAAA-(35)-TTTACA | 35 | 452 | ORF |
| SPy1131 | TGTAAA-(34)-TTTACA | 34 | 480 | ORF |
| SPy0560 | TGTAAA-(8)-TTTACA | 8 | 551 | ORF |
| SPy0073 | TGTAAA-(15)-TTTACA | 15 | 611 | ORF |
| SPy0454 | TGTAAA-(40)-TTTACA | 40 | 721 | ORF |
| SPy0875 | TGTAAA-(22)-TTTACA | 22 | 1187 | ORF |
| 1. ***Streptococcus suis* BM407** |  |  |  |  |
| SSUBM407_1137 | TGTAAA-(30)-TTTACA | 30 | -424 | Prom |
| SSUBM407_1682 | TGTAAA-(19)-TTTACA | 19 | -330 | Prom |
| SSUBM407_0168 | TGTAAA-(13)-TTTACA | 13 | -320 | Prom |
| SSUBM407_0311 | TGTAAA-(40)-TTTACA | 40 | -161 | Prom |
| SSUBM407_1736 | TGTAAA-(28)-TTTACA | 28 | -143 | Prom |
| SSUBM407_0307 | TGTAAA-(22)-TTTACA | 22 | -119 | Prom |
| SSUBM407_1730 | TGTAAA-(22)-TTTACA | 22 | -88 | Prom |
| SSUBM407_0308 | TGTAAA-(22)-TTTACA | 22 | -69 | Prom |
| SSUBM407_0725 | TGTAAA-(3)-TTTACA | 3 | -41 | Prom |
| SSUBM407_0333 | TGTAAA-(18)-TTTACA | 18 | -16 | Prom |
| SSUBM407_0112 | TGTAAA-(13)-TTTACA | 13 | -12 | Prom |
| SSUBM407_0016 | TGTAAA-(17)-TTTACA | 17 | 38 | ORF |
| SSUBM407_0920 | TGTAAA-(15)-TTTACA | 15 | 171 | ORF |
| SSUBM407_1238 | TGTAAA-(29)-TTTACA | 29 | 329 | ORF |
| SSUBM407_0588 | TGTAAA-(16)-TTTACA | 16 | 626 | ORF |
| SSUBM407_0193 | TGTAAA-(30)-TTTACA | 30 | 815 | ORF |
| SSUBM407_1449 | TGTAAA-(31)-TTTACA | 31 | 818 | ORF |
| SSUBM407_1774 | TGTAAA-(18)-TTTACA | 18 | 869 | ORF |
| SSUBM407_0868 | TGTAAA-(32)-TTTACA | 32 | 887 | ORF |
| SSUBM407_1948 | TGTAAA-(19)-TTTACA | 19 | 1127 | ORF |
| 1. ***Streptococcus thermophilus* CNRZ1066** |  |  |  |  |
| str1856 | TGTAAA-(6)-TTTACA | 6 | -106 | Prom |
| str0189 | TGTAAA-(26)-TTTACA | 26 | -94 | Prom |
| str0315 | TGTAAA-(16)-TTTACA | 16 | -6 | Prom |
| str1365 | TGTAAA-(29)-TTTACA | 29 | 11 | ORF |
| str0917 | TGTAAA-(10)-TTTACA | 10 | 534 | ORF |
| str0019 | TGTAAA-(15)-TTTACA | 15 | 538 | ORF |
| str2012 | TGTAAA-(16)-TTTACA | 16 | 653 | ORF |
| str0145 | TGTAAA-(31)-TTTACA | 31 | 1154 | ORF |
| **3. *Staphylococcus*** |  |  |  |  |
| ***Staphylococcus epidermidis* ATCC 12228** |  |  |  |  |
| SE0709 | TGTAAA-(20)-TTTACA | 20 | -498 | Prom |
| SE0184 | TGTAAA-(1)-TTTACA | 1 | -385 | Prom |
| SE0245 | TGTAAA-(0)-TTTACA | 0 | -367 | Prom |
| SE2004 | TGTAAA-(37)-TTTACA | 37 | -220 | Prom |
| SE2196 | TGTAAA-(11)-TTTACA | 11 | -214 | Prom |
| SE0899 | TGTAAA-(25)-TTTACA | 25 | -207 | Prom |
| SE0833 | TGTAAA-(9)-TTTACA | 9 | -188 | Prom |
| SE0900 | TGTAAA-(25)-TTTACA | 25 | -187 | Prom |
| SE2003 | TGTAAA-(37)-TTTACA | 37 | -146 | Prom |
| SE0185 | TGTAAA-(1)-TTTACA | 1 | -118 | Prom |
| SE2197 | TGTAAA-(11)-TTTACA | 11 | -105 | Prom |
| SE0351 | TGTAAA-(17)-TTTACA | 17 | -101 | Prom |
| SE0350 | TGTAAA-(17)-TTTACA | 17 | -94 | Prom |
| SE1722 | TGTAAA-(9)-TTTACA | 9 | -94 | Prom |
| SE0834 | TGTAAA-(9)-TTTACA | 9 | -93 | Prom |
| SE0710 | TGTAAA-(20)-TTTACA | 20 | -91 | Prom |
| SE2395 | TGTAAA-(5)-TTTACA | 5 | -72 | Prom |
| SE1856 | TGTAAA-(17)-TTTACA | 17 | -68 | Prom |
| SE2335 | TGTAAA-(15)-TTTACA | 15 | -45 | Prom |
| SE0344 | TGTAAA-(17)-TTTACA | 17 | -42 | Prom |
| SE0233 | TGTAAA-(17)-TTTACA | 17 | -41 | Prom |
| SE0706 | TGTAAA-(25)-TTTACA | 25 | 53 | ORF |
| SE1072 | TGTAAA-(7)-TTTACA | 7 | 56 | ORF |
| SE2330 | TGTAAA-(33)-TTTACA | 33 | 89 | ORF |
| SE2226 | TGTAAA-(6)-TTTACA | 6 | 92 | ORF |
| SE1326 | TGTAAA-(26)-TTTACA | 26 | 134 | ORF |
| SE2325 | TGTAAA-(0)-TTTACA | 0 | 177 | ORF |
| SE1273 | TGTAAA-(13)-TTTACA | 13 | 200 | ORF |
| SE1018 | TGTAAA-(36)-TTTACA | 36 | 248 | ORF |
| SE1423 | TGTAAA-(16)-TTTACA | 16 | 320 | ORF |
| SE2036 | TGTAAA-(21)-TTTACA | 21 | 326 | ORF |
| SE0720 | TGTAAA-(18)-TTTACA | 18 | 378 | ORF |
| SE1559 | TGTAAA-(28)-TTTACA | 28 | 413 | ORF |
| SE1694 | TGTAAA-(6)-TTTACA | 6 | 425 | ORF |
| SE2009 | TGTAAA-(9)-TTTACA | 9 | 521 | ORF |
| SE1128 | TGTAAA-(31)-TTTACA | 31 | 740 | ORF |
| SE2401 | TGTAAA-(16)-TTTACA | 16 | 806 | ORF |
| SE1073 | TGTAAA-(9)-TTTACA | 9 | 914 | ORF |
| SE1220 | TGTAAA-(23)-TTTACA | 23 | 1134 | ORF |
| SE0624 | TGTAAA-(21)-TTTACA | 21 | 1445 | ORF |
| SE1357 | TGTAAA-(6)-TTTACA | 6 | 1655 | ORF |
| SE2395 | TGTAAA-(16)-TTTACA | 16 | 1772 | ORF |
| **4. *Listeria*** |  |  |  |  |
| ***Listeria monocytogenes* EGD-e** |  |  |  |  |
| lmo2682 | TGTAAA-(1)-TTTACA | 1 | -214 | Prom |
| lmo1125 | TGTAAA-(24)-TTTACA | 24 | -156 | Prom |
| lmo2683 | TGTAAA-(1)-TTTACA | 1 | -139 | Prom |
| lmo0454 | TGTAAA-(36)-TTTACA | 36 | -129 | Prom |
| lmo2004 | TGTAAA-(28)-TTTACA | 28 | -122 | Prom |
| lmo1513 | TGTAAA-(20)-TTTACA | 20 | -105 | Prom |
| lmo2196 | TGTAAA-(5)-TTTACA | 5 | -89 | Prom |
| lmo1797 | TGTAAA-(10)-TTTACA | 10 | -84 | Prom |
| lmo2263 | TGTAAA-(11)-TTTACA | 11 | -82 | Prom |
| lmo2581 | TGTAAA-(17)-TTTACA | 17 | -75 | Prom |
| lmo0455 | TGTAAA-(36)-TTTACA | 36 | -74 | Prom |
| lmo2262 | TGTAAA-(11)-TTTACA | 11 | -59 | Prom |
| lmo0748 | TGTAAA-(17)-TTTACA | 17 | -53 | Prom |
| lmo0350 | TGTAAA-(6)-TTTACA | 6 | -48 | Prom |
| lmo2437 | TGTAAA-(3)-TTTACA | 3 | 38 | ORF |
| lmo1299 | TGTAAA-(9)-TTTACA | 9 | 50 | ORF |
| lmo0844 | TGTAAA-(16)-TTTACA | 16 | 62 | ORF |
| lmo2220 | TGTAAA-(39)-TTTACA | 39 | 68 | ORF |
| lmo0168 | TGTAAA-(16)-TTTACA | 16 | 86 | ORF |
| lmo2779 | TGTAAA-(31)-TTTACA | 31 | 170 | ORF |
| lmo2447 | TGTAAA-(7)-TTTACA | 7 | 188 | ORF |
| lmo0061 | TGTAAA-(33)-TTTACA | 33 | 197 | ORF |
| lmo0281 | TGTAAA-(40)-TTTACA | 40 | 212 | ORF |
| lmo0540 | TGTAAA-(21)-TTTACA | 21 | 251 | ORF |
| lmo0514 | TGTAAA-(36)-TTTACA | 36 | 347 | ORF |
| lmo1862 | TGTAAA-(10)-TTTACA | 10 | 389 | ORF |
| lmo0974 | TGTAAA-(23)-TTTACA | 23 | 434 | ORF |
| lmo1077 | TGTAAA-(22)-TTTACA | 22 | 455 | ORF |
| lmo0945 | TGTAAA-(28)-TTTACA | 28 | 521 | ORF |
| lmo2022 | TGTAAA-(19)-TTTACA | 19 | 695 | ORF |
| lmo1902 | TGTAAA-(40)-TTTACA | 40 | 740 | ORF |
| lmo1103 | TGTAAA-(5)-TTTACA | 5 | 767 | ORF |
| lmo1598 | TGTAAA-(13)-TTTACA | 13 | 788 | ORF |
| lmo1774 | TGTAAA-(34)-TTTACA | 34 | 797 | ORF |
| lmo2487 | TGTAAA-(40)-TTTACA | 40 | 1253 | ORF |
| lmo0627 | TGTAAA-(24)-TTTACA | 24 | 1820 | ORF |
| **5. *Enterococcus*** |  |  |  |  |
| ***Enterococcus faecium* V583** |  |  |  |  |
| EF2740 | TGTAAA-(38)-TTTACA | 38 | -418 | Prom |
| EF0452 | TGTAAA-(2)-TTTACA | 2 | -204 | Prom |
| EF0149 | TGTAAA-(16)-TTTACA | 16 | -203 | Prom |
| EF2750 | TGTAAA-(18)-TTTACA | 18 | -198 | Prom |
| EF0294 | TGTAAA-(31)-TTTACA | 31 | -162 | Prom |
| EF0406 | TGTAAA-(33)-TTTACA | 33 | -141 | Prom |
| EF0464 | TGTAAA-(40)-TTTACA | 40 | -138 | Prom |
| EF0692 | TGTAAA-(33)-TTTACA | 33 | -135 | Prom |
| EF0293 | TGTAAA-(31)-TTTACA | 31 | -127 | Prom |
| EF2710 | TGTAAA-(21)-TTTACA | 21 | -125 | Prom |
| EF2856 | TGTAAA-(10)-TTTACA | 10 | -121 | Prom |
| EF0405 | TGTAAA-(33)-TTTACA | 33 | -102 | Prom |
| EF0693 | TGTAAA-(33)-TTTACA | 33 | -88 | Prom |
| EF3182 | TGTAAA-(12)-TTTACA | 12 | -68 | Prom |
| EF2711 | TGTAAA-(21)-TTTACA | 21 | -56 | Prom |
| EF1048 | TGTAAA-(10)-TTTACA | 10 | -38 | Prom |
| EF0453 | TGTAAA-(2)-TTTACA | 2 | -30 | Prom |
| EF1153 | TGTAAA-(20)-TTTACA | 20 | -9 | Prom |
| EF1151 | TGTAAA-(33)-TTTACA | 33 | -7 | Prom |
| EFA0001 | TGTAAA-(5)-TTTACA | 5 | 23 | ORF |
| EF1589 | TGTAAA-(9)-TTTACA | 9 | 24 | ORF |
| EF0915 | TGTAAA-(37)-TTTACA | 37 | 38 | ORF |
| EF3160 | TGTAAA-(22)-TTTACA | 22 | 48 | ORF |
| EF3178 | TGTAAA-(27)-TTTACA | 27 | 53 | ORF |
| EFA0080 | TGTAAA-(4)-TTTACA | 4 | 135 | ORF |
| EF2213 | TGTAAA-(7)-TTTACA | 7 | 152 | ORF |
| EF1620 | TGTAAA-(36)-TTTACA | 36 | 185 | ORF |
| EF1875 | TGTAAA-(34)-TTTACA | 34 | 188 | ORF |
| EF0163 | TGTAAA-(34)-TTTACA | 34 | 212 | ORF |
| EF1646 | TGTAAA-(16)-TTTACA | 16 | 239 | ORF |
| EF0643 | TGTAAA-(35)-TTTACA | 35 | 306 | ORF |
| EF1783 | TGTAAA-(33)-TTTACA | 33 | 333 | ORF |
| EF1221 | TGTAAA-(14)-TTTACA | 14 | 413 | ORF |
| EF_C0006 | TGTAAA-(14)-TTTACA | 14 | 930 | ORF |
| EF1646 | TGTAAA-(30)-TTTACA | 30 | 1217 | ORF |
| EF0348 | TGTAAA-(25)-TTTACA | 25 | 1220 | ORF |
| **6. *Lactobacillus*** |  |  |  |  |
| 1. ***Lactobacillus casei* ATCC 334** |  |  |  |  |
| LSEI_0728 | TGTAAA-(18)-TTTACA | 18 | -418 | Prom |
| LSEI_0226 | TGTAAA-(3)-TTTACA | 3 | -206 | Prom |
| LSEI_1332 | TGTAAA-(23)-TTTACA | 23 | -196 | Prom |
| LSEI_2271 | TGTAAA-(27)-TTTACA | 27 | -164 | Prom |
| LSEI_2270 | TGTAAA-(27)-TTTACA | 27 | -163 | Prom |
| LSEI_0752 | TGTAAA-(19)-TTTACA | 19 | -134 | Prom |
| LSEI_0729 | TGTAAA-(18)-TTTACA | 18 | -116 | Prom |
| LSEI_0227 | TGTAAA-(3)-TTTACA | 3 | -111 | Prom |
| LSEI_1601 | TGTAAA-(10)-TTTACA | 10 | -79 | Prom |
| LSEI_2516 | TGTAAA-(10)-TTTACA | 10 | -72 | Prom |
| 1. ***Lactobacillus delbrueckii* subsp. *bulgaricus* ATCC BAA 365** |  |  |  |  |
| LBUL_1552 | TGTAAA-(8)-TTTACA | 8 | 745 | ORF |
| LBUL_1818 | TGTAAA-(32)-TTTACA | 32 | 129 | ORF |
| 1. ***Lactobacillus plantarum* WCFS1** |  |  |  |  |
| pWCFS102_04 | TGTAAA-(37)-TTTACA | 37 | -332 | Prom |
| lp_2516 | TGTAAA-(12)-TTTACA | 12 | -263 | Prom |
| lp_2499 | TGTAAA-(21)-TTTACA | 21 | -208 | Prom |
| lp_0439 | TGTAAA-(12)-TTTACA | 12 | -183 | Prom |
| lp_0717 | TGTAAA-(5)-TTTACA | 5 | -181 | Prom |
| lp_1175 | TGTAAA-(17)-TTTACA | 17 | -154 | Prom |
| lp_0715 | TGTAAA-(5)-TTTACA | 5 | -115 | Prom |
| lp_3018 | TGTAAA-(15)-TTTACA | 15 | -90 | Prom |
| lp_3019 | TGTAAA-(15)-TTTACA | 15 | -50 | Prom |
| lp_3626 | TGTAAA-(7)-TTTACA | 7 | 134 | ORF |
| lp_3117 | TGTAAA-(17)-TTTACA | 17 | 692 | ORF |
| lp_2901 | TGTAAA-(14)-TTTACA | 14 | 977 | ORF |

^a^The position of the leftmost base"T" within the left 6-bp core sequence (TGTAAA) relative to the respective translation start point of the associated gene.

^b^Prom:promoter region; ORF:open reading frame.
