# Supplementary material for: A Flexible Binding Site Architecture Provides New Insights into CcpA Global Regulation in Gram-Positive Bacteria
Source: mBio. 2017 Jan 24;8(1):e02004-16. doi: 10.1128/mBio.02004-16 (PMC5263246; doi:10.1128/mBio.02004-16)
Supplement: TABLE S4 [file mbo002173155st4.docx]

**Table S4. Strains and plasmids used in this study.**

| Strains or plasmids | Description of genotype | Source or reference |
| --- | --- | --- |
| **Bacterial strains** |  |  |
| *C. acetobutylicum* |  |  |
| 824 | Wild type | ATCC |
| 824ccpA | *ccpA*::intron | ([1](#_ENREF_1)) |
| 824-lacZ | ATCC 824, carries the pIMP1-lacZ plasmid | This study |
| 824-P*_sol_*-lacZ | ATCC 824, carries the pIMP1-P*_sol_***-**lacZ plasmid | This study |
| 824-P*_sol_*-Mu1-lacZ | ATCC 824, carries the pIMP1- P*_sol_*_-Mu1_-lacZ plasmid | This study |
| 824-P*_sol_*-Mu2-lacZ | ATCC 824, carries the pIMP1- P*_sol_*_-Mu2_-lacZ plasmid | This study |
| 824-P*_sol_*-Mu12-lacZ | ATCC 824, carries the pIMP1- P*_sol_*_-Mu12_-lacZ plasmid | This study |
| 824-L1-lacZ | ATCC 824, carries the pIMP1-P*_sol_*_-Mu-L1_-lacZ plasmid | This study |
| 824-L2-lacZ | ATCC 824, carries the pIMP1-P*_sol_*_-Mu-L2_-lacZ plasmid | This study |
| 824-L3-lacZ | ATCC 824, carries the pIMP1-P*_sol_*_-Mu-L3_-lacZ plasmid | This study |
| 824-L4-lacZ | ATCC 824, carries the pIMP1-P*_sol_*_-Mu-L4_-lacZ plasmid | This study |
| 824-L5-lacZ | ATCC 824, carries the pIMP1-P*_sol_*_-Mu-L5_-lacZ plasmid | This study |
| 824-L6-lacZ | ATCC 824, carries the pIMP1-P*_sol_*_-Mu-L6_-lacZ plasmid | This study |
| 824-R1-lacZ | ATCC 824, carries the pIMP1-P*_sol_*_-Mu-R1_-lacZ plasmid | This study |
| 824-R2-lacZ | ATCC 824, carries the pIMP1-P*_sol_*_-Mu-R2_-lacZ plasmid | This study |
| 824-R3-lacZ | ATCC 824, carries the pIMP1-P*_sol_*_-Mu-R3_-lacZ plasmid | This study |
| 824-R4-lacZ | ATCC 824, carries the pIMP1-P*_sol_*_-Mu-R4_-lacZ plasmid | This study |
| 824-R5-lacZ | ATCC 824, carries the pIMP1-P*_sol_*_-Mu-R5_-lacZ plasmid | This study |
| 824-R6-lacZ | ATCC 824, carries the pIMP1-P*_sol_*_-Mu-R6_-lacZ plasmid | This study |
| 824-P*_cac0804-15_* _(wt)_-lacZ | ATCC 824, carries the pIMP1-P*_cac0804-15_* _(wt)_-lacZ plasmid | This study |
| 824-P*_cac0804-10_*-lacZ | ATCC 824, carries the pIMP1-P*_cac0804-10_*-lacZ plasmid | This study |
| 824-P*_cac0804-6_*-lacZ | ATCC 824, carries the pIMP1-P*_cac0804-6_*-lacZ plasmid | This study |
| 824-P*_cac0804-mu_*-lacZ | ATCC 824, carries the pIMP1-P*_cac0804-mu_*-lacZ plasmid | This study |
| 824ccpA-lacZ | 824ccpA, carries the pIMP1-lacZ plasmid | This study |
| 824ccpA-P*_sol_*-lacZ | 824ccpA, carries the pIMP1-P*_sol_***-**lacZ plasmid | This study |
| 824ccpA-P*_sol_*-Mu1-lacZ | 824ccpA, carries the pIMP1-P*_sol_*_-Mu1_-lacZ plasmid | This study |
| 824ccpA-P*_sol_*-Mu2-lacZ | 824ccpA, carries the pIMP1-P*_sol_*_-Mu2_-lacZ plasmid | This study |
| 824ccpA-P*_sol_*-Mu12-lacZ | 824ccpA, carries the pIMP1-P*_sol_*_-Mu12_-lacZ plasmid | This study |
| 824ccpA-L1-lacZ | 824ccpA, carries the pIMP1-P*_sol_*_-Mu-L1_-lacZ plasmid | This study |
| 824ccpA-L2-lacZ | 824ccpA, carries the pIMP1-P*_sol_*_-Mu-L2_-lacZ plasmid | This study |
| 824ccpA-L3-lacZ | 824ccpA, carries the pIMP1-P*_sol_*_-Mu-L3_-lacZ plasmid | This study |
| 824ccpA-L4-lacZ | 824ccpA, carries the pIMP1-P*_sol_*_-Mu-L4_-lacZ plasmid | This study |
| 824ccpA-L5-lacZ | 824ccpA, carries the pIMP1-P*_sol_*_-Mu-L5_-lacZ plasmid | This study |
| 824ccpA-L6-lacZ | 824ccpA, carries the pIMP1-P*_sol_*_-Mu-L6_-lacZ plasmid | This study |
| 824ccpA-R1-lacZ | 824ccpA, carries the pIMP1-P*_sol_*_-Mu-R1_-lacZ plasmid | This study |
| 824ccpA-R2-lacZ | 824ccpA, carries the pIMP1-P*_sol_*_-Mu-R2_-lacZ plasmid | This study |
| 824ccpA-R3-lacZ | 824ccpA, carries the pIMP1-P*_sol_*_-Mu-R3_-lacZ plasmid | This study |
| 824ccpA-R4-lacZ | 824ccpA, carries the pIMP1-P*_sol_*_-Mu-R4_-lacZ plasmid | This study |
| 824ccpA-R5-lacZ | 824ccpA, carries the pIMP1-P*_sol_*_-Mu-R5_-lacZ plasmid | This study |
| 824ccpA-R6-lacZ | 824ccpA, carries the pIMP1-P*_sol_*_-Mu-R6_-lacZ plasmid | This study |
| 824ccpA-P*_cac0804-15_* _(wt)_-lacZ | 824ccpA, carries the pIMP1-P*_cac0804-15_* _(wt)_-lacZ plasmid | This study |
| 824ccpA-P*_cac0804-10_*-lacZ | 824ccpA, carries the pIMP1-P*_cac0804-10_*-lacZ plasmid | This study |
| 824ccpA-P*_cac0804-6_*-lacZ | 824ccpA, carries the pIMP1-P*_cac0804-6_*-lacZ plasmid | This study |
| 824ccpA-P*_cac0804-mu_*-lacZ | 824ccpA, carries the pIMP1-P*_cac0804-mu_*-lacZ plasmid | This study |
| *E. coli* |  |  |
| Top10 | General cloning host strain | Invitrogen |
| ER2275 | Strain used to methylate the vector | New England Biolabs |
| Rosetta (DE3) | *F^-^ ompT hsdSB(RB^-^ mB^-^) gal dcm λ(DE3 [lacI*  *lacUV5-T7gene 1 ind1 sam7 nin5]) pLysSRARE (CamR)* | Novagen |
| **Plasmids** |  |  |
| pMD-18T | TA-cloning vector | Takara |
| pET-28a | Vector used for protein purification | Invitrogen |
| pET-28a-ccpAcac | pET-28a carrying *ccpA* (CAC3037) from *C. acetobutylicum* | This study |
| pET-28a-ccpAbsu | pET-28a carrying *ccpA* (BSU29740) from *Bacillus subtilis* | This study |
| pET-28a-ccpAcpf | pET-28a carrying *ccpA* (CPF2863) from *Clostridium perfringens* ATCC 13124 | This study |
| pET-28a-HPrK | pET-28a carrying *Hprk* (CAC1820) from *C. acetobutylicum* | ([2](#_ENREF_2)) |
| pGEX4T1-HPr | *Amp^r^*, pGEX4T-1 carrying *Hpr* (CAC1089) from *C. acetobutylicum* | ([2](#_ENREF_2)) |
| placZFT | Vector with *lacZ* reporter gene | ([3](#_ENREF_3)) |
| pIMP1-lacZ | Vector used for β-Galactosidase assay, derived from placZFT, *Cm^r^* | This study |
| pIMP1-P*_sol_*-lacZ | *lacZ* reporter gene driven by *sol* operon promoter | This study |
| pIMP1-P*_sol_*_-Mu1_-lacZ | *lacZ* reporter gene driven by mutated *sol* promoter promoter | This study |
| pIMP1-P*_sol_*_-Mu2_-lacZ | *lacZ* reporter gene driven by mutated *sol* promoter promoter | This study |
| pIMP1-P*_sol_*_-Mu12_-lacZ | *lacZ* reporter gene driven by mutated *sol* promoter promoter | This study |
| pIMP1-P*_sol_*_-Mu-L1_-lacZ | *lacZ* reporter gene driven by mutated *sol* promoter promoter | This study |
| pIMP1-P*_sol_*_-Mu-L2_-lacZ | *lacZ* reporter gene driven by mutated *sol* promoter promoter | This study |
| pIMP1-P*_sol_*_-Mu-L3_-lacZ | *lacZ* reporter gene driven by mutated *sol* promoter promoter | This study |
| pIMP1-P*_sol_*_-Mu-L4_-lacZ | *lacZ* reporter gene driven by mutated *sol* promoter promoter | This study |
| pIMP1-P*_sol_*_-Mu-L5_-lacZ | *lacZ* reporter gene driven by mutated *sol* promoter promoter | This study |
| pIMP1-P*_sol_*_-Mu-L6_-lacZ | *lacZ* reporter gene driven by mutated *sol* promoter promoter | This study |
| pIMP1-P*_sol_*_-Mu-R1_-lacZ | *lacZ* reporter gene driven by mutated *sol* promoter promoter | This study |
| pIMP1-P*_sol_*_-Mu-R2_-lacZ | *lacZ* reporter gene driven by mutated *sol* promoter promoter | This study |
| pIMP1-P*_sol_*_-Mu-R3_-lacZ | *lacZ* reporter gene driven by mutated *sol* promoter promoter | This study |
| pIMP1-P*_sol_*_-Mu-R4_-lacZ | *lacZ* reporter gene driven by mutated *sol* promoter promoter | This study |
| pIMP1-P*_sol_*_-Mu-R5_-lacZ | *lacZ* reporter gene driven by mutated *sol* promoter promoter | This study |
| pIMP1-P*_sol_*_-Mu-R6_-lacZ | *lacZ* reporter gene driven by mutated *sol* promoter promoter | This study |
| pIMP1-P*_cac0804-15_* _(wt)_-lacZ | *lacZ* reporter gene driven by P*_cac0804-15_* _(wt)_ | This study |
| pIMP1-P*_cac0804-10_*-lacZ | *lacZ* reporter gene driven by P*_cac0804-10_* | This study |
| pIMP1-P*_cac0804-6_*-lacZ | *lacZ* reporter gene driven by P*_cac0804-6_* | This study |
| pIMP1-P*_cac0804-mu_*-lacZ | *lacZ* reporter gene driven by P*_cac0804-mu_* | This study |

**REFERENCES**

1. **Ren C, Gu Y, Hu S, Wu Y, Wang P, Yang Y, Yang C, Yang S, Jiang W.** 2010. Identification and inactivation of pleiotropic regulator CcpA to eliminate glucose repression of xylose utilization in *Clostridium acetobutylicum*. Metab Eng **12:**446-454.

2. **Wu Y, Yang Y, Ren C, Yang C, Yang S, Gu Y, Jiang W.** 2015. Molecular modulation of pleiotropic regulator CcpA for glucose and xylose coutilization by solvent-producing *Clostridium acetobutylicum*. Metab Eng **28:**169-179.

3. **Feustel L, Nakotte S, Durre P.** 2004. Characterization and Development of Two Reporter Gene Systems for *Clostridium acetobutylicum*. Appl Environ Microbiol **70:**798-803.
